# A chromosome-scale assembly of the quinoa genome provides insights into the structure and dynamics of its subgenomes

**DOI:** 10.1038/s42003-023-05613-4

**Published:** 2023-12-13

**Authors:** Elodie Rey, Peter J. Maughan, Florian Maumus, Daniel Lewis, Leanne Wilson, Juliana Fuller, Sandra M. Schmöckel, Eric N. Jellen, Mark Tester, David E. Jarvis

**Affiliations:** 1 1King Abdullah University of Science and Technology (KAUST), Biological and Environmental Sciences & Engineering Division (BESE), Thuwal, 23955-6900 Saudi Arabia; 2 Brigham Young University, Department of Plant and Wildlife Sciences, College of Life Sciences, Provo, UT 84602 USA; 3 URGI, INRA, Université Paris-Saclay, 78026 Versailles, France; 4 University of Hohenheim, Institute of Crop Science, Department Physiology of Yield Stability, 70599 Stuttgart, Germany

**Keywords:** Polyploidy in plants, Plant evolution, Comparative genomics

## Abstract

Quinoa (*Chenopodium quinoa* Willd.) is an allotetraploid seed crop with the potential to help address global food security concerns. Genomes have been assembled for four accessions of quinoa; however, all assemblies are fragmented and do not reflect known chromosome biology. Here, we use in vitro and in vivo Hi-C data to produce a chromosome-scale assembly of the Chilean accession PI 614886 (QQ74). The final assembly spans 1.326 Gb, of which 90.5% is assembled into 18 chromosome-scale scaffolds. The genome is annotated with 54,499 protein-coding genes, 96.9% of which are located on the 18 largest scaffolds. We also report an updated genome assembly for the B-genome diploid *C. suecicum* and use it, together with the A-genome diploid *C. pallidicaule*, to identify genomic rearrangements within the quinoa genome, including a large pericentromeric inversion representing 71.7% of chromosome Cq3B. Repetitive sequences comprise 65.2%, 48.6%, and 57.9% of the quinoa, *C. pallidicaule*, and *C. suecicum* genomes, respectively. Evidence suggests that the B subgenome is more dynamic and has expanded more than the A subgenome. These genomic resources will enable more accurate assessments of genome evolution within the *Amaranthaceae* and will facilitate future efforts to identify variation in genes underlying important agronomic traits in quinoa.

## Abstract (web summary)

An updated, chromosome-scale genome assembly of quinoa enables the characterization of subgenome dynamics, including the identification of large structural rearrangements. The B subgenome is also more dynamic and has expanded more than the A subgenome.

## Introduction

Quinoa (*Chenopodium quinoa* Willd., 2*n* = 4*x* = 36) has emerged as a nutritious seed crop that has become a trendy health food option in many developed nations. The beneficial aspects of quinoa seeds—including an optimal balance of essential amino acids and a high total protein content compared to common cereals such as rice and maize^1^—also make quinoa an attractive crop for nations that are more susceptible to malnutrition and food security issues^2^. For this reason, quinoa production has recently increased throughout the world, although most quinoa is still produced near its center of origin in the Andean region of South America^3^. Quinoa is well adapted to growth in this region, with highland accessions that are tolerant of the cold and high UV light characteristic of the high Andean mountains and plains of Bolivia, Peru, and Ecuador, and coastal accessions that are better adapted to the warmer and more humid climates of coastal Chile. However, quinoa is not well adapted to the biotic and abiotic conditions encountered outside these regions of South America, including lowland tropical, subtropical, and warm-season temperate production environments^4^. Quinoa is also not well adapted to high-throughput agricultural production systems and suffers from undesirable agronomic traits such as lodging, pre-harvest sprouting, seed shattering, and susceptibility to novel pests and diseases^4^.

The development of genetic and genomic resources for quinoa will accelerate its improvement for enhanced growth in new production systems throughout the world. Genome assemblies have been produced for four quinoa accessions to date: Kd^5^, an accession inbred for more than 20 years in Japan; PI 614886 (QQ74)^6^, a coastal Chilean accession; Real^7^, a highland accession representing an ecotype that is one of the most common commercial varieties; and an assembly based primarily on the Bolivian accession CHEN125^8^. The assembly of PI 614886 was the most contiguous of the three and was produced using a combination of PacBio sequencing, Bionano optical maps, and in vitro Chicago Hi-C. This assembly contained 3,486 scaffolds spanning 1.385 Gb; anchoring the assembly to a linkage map resulted in 18 scaffolds spanning 1.183 Gb. Genome assemblies were also produced for two diploid species representing the two subgenomes in quinoa: the A-genome diploid *C. pallidicaule* and the B-genome diploid *C. suecicum*^6^. Sequencing these diploid species enabled the identification of nine quinoa chromosomes belonging to the A subgenome and nine belonging to the B subgenome, and analysis of the rate of synonymous substitutions per synonymous site (Ks) in pairs of homoeologous genes in quinoa indicated that the original hybridization between ancestral A- and B-genome species occurred 3.3–6.3 million years ago.

Despite the utility of these initial genome sequences, the assemblies were incomplete and not chromosome-scale. For example, the 18 quinoa pseudomolecules produced by anchoring scaffolds to the genetic map only contained 85% of the total assembly, and the broad range of chromosome sizes—ranging from 137.41 to 14.69 Mb—didn’t match cytogenetic evidence^9^. Furthermore, the *C. pallidicaule* and *C. suecicum* assemblies were produced using short-read sequencing data and consisted of several thousand contigs each. We recently produced a chromosome-scale assembly of *C. pallidicaule*^10^, and here we present an improved assembly of *C. suecicum* and a chromosome-scale assembly of quinoa using Hi-C. Hi-C is a high-throughput approach that identifies physical contacts between DNA fragments in their in vivo chromosomal conformation. DNA fragments that contact each other at high frequency are inferred to be physically closer on a chromosome than DNA fragments that contact each other at lower frequency, and this information can be used to order and orient sequencing contigs into scaffolds, often at or near chromosome scale lengths^11,12^.

Hi-C can be used to scaffold contig-level assemblies produced from either short- or long-read sequencing data alone^13^, or to further scaffold these contig-level assemblies after initial scaffolding with other technologies such as optical maps or in vitro Hi-C^14^. In our efforts to improve the assembly of quinoa accession PI 614886, we sought to identify which technologies used to produce the input assembly resulted in the best output assembly when scaffolded with in vivo Hi-C. Specifically, we used a PacBio-only assembly or PacBio assemblies initially scaffolded with either Bionano optical maps or in vitro Chicago Hi-C as input assemblies to be scaffolded with in vivo Hi-C. We found that all three output assemblies represented substantial improvement over the previously reported assembly that was scaffolded with Bionano optical maps and Chicago in vitro Hi-C maps and anchored to a genetic linkage map^6^. The combination of in vitro and in vivo Hi-C produced the best output assembly by several measures. This final assembly contains 90.5% of the 1.326 Gb assembly in 18 large scaffolds, corresponding to the haploid quinoa chromosomes. Using this assembly, we performed gene and TE annotations to support synteny analyses and provide first insights into the structure and dynamic of the quinoa subgenomes.

## Results

### Genome assembly, annotation, and assessment

We previously produced multiple genome assemblies of quinoa accession PI 614886 (Supplementary Fig. 1): a contig-level assembly produced using PacBio (PB); or scaffold-level assemblies produced using the PacBio assembly scaffolded with Bionano optical maps alone (PB+BN), Bionano optical maps plus in vitro Chicago Hi-C (PB+BN+Chi), or Bionano optical maps plus in vitro Chicago Hi-C and a linkage map (PB+BN+Chi+linkage)^6^. To improve the quinoa assembly, we generated 175 million in vivo Hi-C read pairs and used them to scaffold three input assemblies (Supplementary Fig. 1): the previously reported PB and PB + BN assemblies, and a new assembly produced using the PB assembly scaffolded with in vitro Chicago Hi-C (PB+Chi). The PB+Chi assembly was the most contiguous of the three input assemblies, consisting of 3,127 scaffolds with a scaffold N50 of 11.75 Mb (Supplementary Table 1). When scaffolded with in vivo Hi-C, all three output assemblies showed dramatic improvements in contiguity (Supplementary Table 1, Supplementary Fig. 2). All three output assemblies were chromosome-scale, with each containing 18 major scaffolds corresponding to the 18 haploid chromosomes (Supplementary Fig. 2). The PB+BN assembly contained the longest scaffold and the largest scaffold N50 value of all three assemblies; however, this assembly contained more total scaffolds and had a slightly lower percentage of the total assembly contained in the 18 largest scaffold (Supplementary Table 1). The PB and PB+Chi assemblies each incorporated 90.5% of the input sequence length into the 18 largest chromosomes, although the PB+Chi assembly had a slightly higher percentage (90.25%) of the total sequence length in scaffolded contigs with high-differential log-likelihood scores than the PB (89.04%) and PB+BN (88.16%) assemblies (Supplementary Table 2). These results indicate that the PB+Chi input assembly produced the most contiguous, highest-quality assembly when scaffolded with Hi-C.

To provide further support for the selection of the best assembly, we analyzed gene collinearity between homoeologous chromosomes in each assembly. We reasoned that the most correct assembly would be the one that displayed the highest degree of subgenome collinearity. Subgenome assignments were previously made for each chromosome in the previously reported assembly of QQ74 anchored to a genetic linkage map (hereafter, V1 assembly)^6^. To identify homoeologous chromosomes belonging to the A and B subgenomes in the new Hi-C assemblies, we first transferred the gene annotations from the V1 assembly to all three Hi-C assemblies and then identified chromosomes that were syntenic to the V1 chromosomes (Supplementary Fig. 3). From this comparison, we identified nine A subgenome chromosomes and nine B subgenome chromosomes. As expected, given the broad range of chromosomes sizes in the V1 assembly, we found that some of the largest chromosomes—such as Chr1B and Chr7A—were misassemblies and represented multiple chromosomes. Likewise, some of the smallest chromosomes—such as Chr9B and Chr13A—represented only portions of larger chromosomes. In the Hi-C assemblies, each set of homoeologous chromosomes identified by comparison to the V1 linkage assembly showed a 2:1 syntenic relationship with a single *Beta vulgaris* (sugar beet) chromosome (Supplementary Fig. 4), indicating that the subgenome assignments were correct. Thus, chromosomes in each Hi-C assembly were named based on their subgenome assignment and their homologous relationship to the nine *B. vulgaris* chromosomes.

Having identified the homoeologous chromosomes in each Hi-C assembly, we compared the positions of homoeologous gene pairs in order to identify the assembly with the highest degree of subgenome synteny (Supplementary Fig. 5). All three Hi-C assemblies showed a higher percentage of collinear genes in the subgenomes than the original V1 assembly (Supplementary Table 3). The PB+BN Hi-C assembly showed the lowest degree of collinearity (Supplementary Fig. 5, Supplementary Table 3), suggesting that it contains the most misassembled regions. The PB+Chi Hi-C assembly showed the highest degree of collinearity within its subgenomes and with *B. vulgaris* (Supplementary Table 3).

Finally, we assessed the quality of the PB, PB+BN, and PB+Chi Hi-C assemblies by analyzing the physical positions of mapped SNP markers. We previously created a linkage map from SNPs identified in RNA-seq data from a biparental population of quinoa varieties Kurmi and 0654^6^, and we compared the genetic positions of these markers to their physical positions in each assembly. Although the linkage map contained more linkage groups (26) than expected (18), there was a clear correspondence between physical and genetic positions in all three assemblies; however, the PB+BN assembly showed dramatically less agreement between physical and genetic positions than the PB and PB+Chi assemblies, which were almost identical to each other (Supplementary Fig. 6). No markers were found on Chr7A in any of the assemblies. This chromosome was incorrectly assembled with Chr3A into a single mosaic chromosome in the previously reported V1 assembly (Supplementary Fig. 3).

**Fig. 1:**
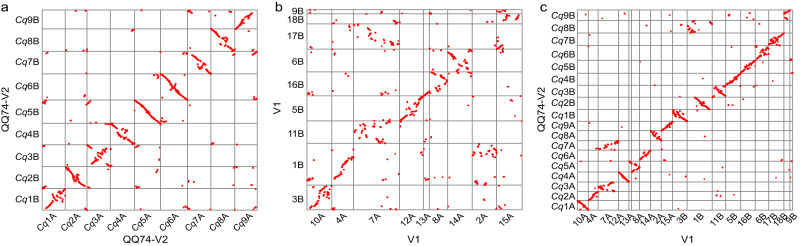
Collinear gene pairs. **a**, **b** Collinearity between subgenomes within the QQ74-V2 (**a**) and V1 (**b**) assemblies. **c** Collinearity between the QQ74-V2 and V1 assemblies.

Together, these analyses suggest that the PB+Chi input assembly produced the most correct Hi-C scaffolded assembly, as it was the most contiguous and showed the highest degree of gene collinearity within its subgenomes and compared to *B. vulgaris*. We therefore performed gap-filling of this assembly using PacBio sequencing reads and subsequently polished the assembly using Illumina reads. We refer to this final assembly as the quinoa QQ74-V2 assembly and use it in all analyses hereafter. Gap-filling and polishing resulted in only very minor changes to the PB+Chi Hi-C assembly (Table 1). To verify that no major structural changes were introduced during gap-filling and polishing, we transferred the gene annotations from the V1 assembly to this final QQ74-V2 assembly and again assessed self-synteny between the homoeologous chromosomes. We found a much higher degree of subgenome synteny in this final assembly (Fig. 1a) than in the original V1 assembly (Fig. 1b). We again identified major structural differences between the original V1 assembly and the new QQ74-V2 assembly, many of which were due to incorrect joining of chromosomes or failure to correctly join chromosomes in the V1 assembly (Fig. 1c).

**Table 1:** Assembly statistics of the PB+Chi Hi-C assembly before and after gap-filling and polishing.

	PB+Chi	PB+Chi +Gap-filling	PB+Chi +Gap-filling +Polishing (QQ74-V2)
Total assembly size (bp)	1,326,352,420	1,326,464,434	1,326,337,151
Number of scaffolds	2942	2942	2942
Longest scaffold (bp)	87,286,765	87,293,964	87,284,847
Scaffold N50 (bp)	66,922,838	66,929,873	66,924,138
Scaffold L50	9	9	9
Scaffold N90 (bp)	53,754,609	53,756,300	53,750,966
Scaffold L90	18	18	18
Percentage in 18 largest scaffolds	90.5	90.5	90.5

We used several approaches to assess the completeness and contiguity of the V1 and QQ74-V2 assemblies (Table 2). First, we identified universal single-copy orthologous genes using BUSCO. Similar numbers of BUSCO genes were identified in the QQ74-V2 and the original V1[6] assemblies, two of which were fragmented in the QQ74-V2 assembly and one in the V1 assembly (Table 2). Slightly more of the complete BUSCO genes were found to be duplicated in the QQ74-V2 assembly (1,298, 80.4%) than in the V1 assembly (1,261, 78.1%), indicating that misassemblies in the V1 assembly prevented the identification of some homoeologous gene pairs in the tetraploid genome (Table 2). Next, we calculated the LTR Assembly Index (LAI)^15^ by evaluating intact and total interspersed Long Terminal Repeat (LTR) elements in both assemblies. LAI scores of 19.15 and 19.44 for the V1 and QQ74-V2 genomes assemblies, respectively, categorize both assemblies as “reference sequence”. We also assessed k-mer completeness and base-level consensus quality (QV) using Merqury^16^ with a set of 215 M (~30×) PE sequencing reads previously published with the V1 reference assembly. K-mer completeness improved substantially from the V1 (90.01%) to QQ74-V2 (98.61%) assembly, while QV values were high for both assemblies (35.31 for the V1 and 36.95 for QQ74-V2, where Q30 corresponds to 99.9% accuracy). Finally, we assessed the read mapping rate by mapping the Illumina sequencing reads to each reference assembly using bowtie2^17^ with sensitive mapping parameters leveraged to retrieve mapping results with low mismatches. The mapping rate increased substantially from 96.43% to 99.06% in the V1 to QQ74-V2 assemblies, respectively. Together, these results show the improvement in sequence contiguity and completeness from the V1 to QQ74_V2 assemblies. All three analyses were also conducted for the *C. suecicum* V2 assembly (Table 2).

**Table 2:** Completeness assessment of the V1, QQ74-V2, and *C. suecicum* V2 genome assemblies.

	PB + BN+Chi	QQ74-V2	*C. suecicum* V2
Complete BUSCOs (%)	1589 (98.4)	1588 (98.4)	1571 (97.3)
Complete and single-copy BUSCOs (%)	328 (20.3)	290 (18.0)	1550 (96.0)
Complete and duplicated BUSCOs (%)	1261 (78.1)	1298 (80.4)	21 (1.3)
Fragmented BUSCOs (%)	1 (0.1)	2 (0.1)	10 (0.6)
Missing BUSCOs (%)	24 (1.5)	24 (1.5)	33 (2.1)
Total BUSCO groups searched	1614	1614	1614
LAI	19.1	19.4	8.7
K-mer completeness (%)	90.0	98.6	90.8
Consensus quality value (QV)	35.3	36.9	29.1
Illumina reads mapping rate (%)	96.4	99.0	93.9

**Fig. 2:**
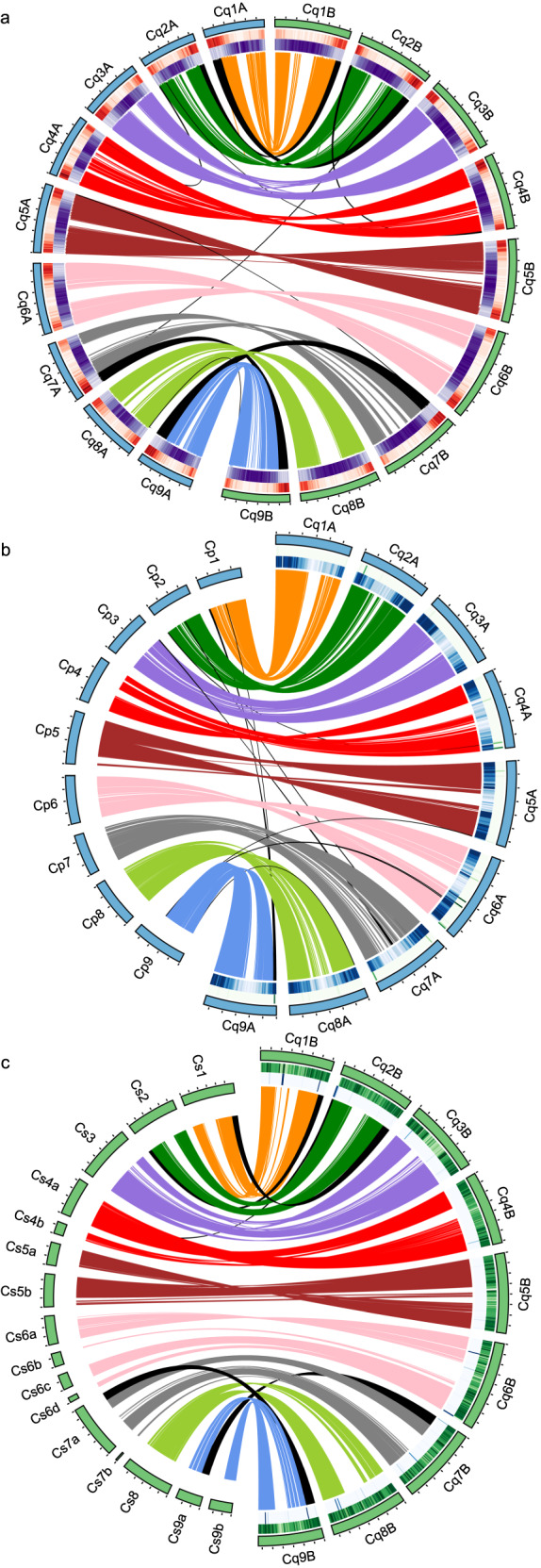
Genome structure and gene collinearity. Each colored link represents a block of collinear genes between orthologous chromosomes; black links represent collinearity between non-orthologous chromosomes. **a** Structure of quinoa chromosomes and collinearity relationships between quinoa subgenomes. From outside to inside, tracks represent chromosome names, positions (Mb), chromosome ideograms (blue, A subgenome; green, B subgenome), gene density in 1-Mb windows (with lowest to highest density expressed as a gradient of intensity from white to red), and TE density in 1-Mb windows (with lowest to highest density expressed as a gradient of intensity from white to purple). **b**, **c** Chromosome structure and collinearity between the quinoa A subgenome and *C. pallidicaule* (**b**) and between quinoa B subgenome and *C. suecicum* (**c**). From outside to inside, tracks represent chromosome names, positions (Mb), chromosome ideograms (blue, A genomes; green, B genomes), *C. suecicum* short-reads mapping rate in 1-Mb windows, and *C. pallidicaule* short-reads mapping rate in 1-Mb windows.

A new annotation of quinoa protein-coding genes was performed based on the QQ74-V2 assembly using MAKER2 and previously reported quinoa RNA-seq and PacBio isoform sequencing (Iso-seq) datasets^6^ representing the transcripts pool from eight tissues, including apical meristems, lateral meristems, whole seedlings, flowers and immature seeds, leaves petioles, stems, shoots, and roots. The new annotation contains 54,499 protein-coding gene models, of which 96.9% (2.7% more than in V1) are on the 18 chromosome-scale scaffolds (Table 3). With 59,071,539 bp of the total coding region, the genic space accounts for 4.45% of the total assembly size and 4.07% of the 1.45 Gb estimated genome size^18,19^ [16,17]. Gene distribution, which shows increasing density toward the telomeres, indicated that the structure of QQ74-V2 pseudomolecules is in better agreement with cytogenetic observations than the V1 assembly (Fig. 2a). A total of 30,458 (55.9%) genes has an annotation edit distance (AED) value ≤ 0.3 (Supplementary Fig. 7). A higher percentage of complete BUSCOs were identified in the QQ74-V2 annotation (96.4%) than in the V1 annotation (95.4%) (Supplementary Table 4). The QQ74-V2 annotation also had slightly fewer fragmented and missing genes (Supplementary Table 4). We assessed the similarity between the V1 and QQ74-V2 annotations by comparing gene models in each annotation using GffCompare (Supplementary Table 5). Of the 43,649 gene models transferred from the V1 to QQ74-V2 pseudomolecules, 38,464 (88.1%) found a match with at least one QQ74-V2 gene model, out of which 10,145 found a complete, exact match of intron chain (same introns at the exact same coordinates). We identified 5,212 V1 genes that are missing on QQ74-V2 pseudomolecules and 10,167 genes that were newly annotated in QQ74-V2 with an average annotation edit score (AED) of 0.34. As a resource to compare genes across assembly versions, we have created a correspondence table that links gene IDs from the V1 and QQ74-V2 annotations (Supplementary Data 1).

**Table 3:** Gene annotation statistics for the V1, QQ74-V2, and *C. suecicum* V2 assemblies.

	V1	QQ74-V2	*C. suecicum* V2
Total number of protein-coding genes	44,776	54,499	29,702
Number of genes on anchored chromosomes (%)	42,240 (94.3)	52,856 (96.9)	29,436 (99)
Number of genes with annotation edit distance (AED) ≤ 0.3	33,365	30,458	21,057
Total coding region (bp)	57,064,233	59,071,539	33,621,006
Average gene length (bp)	4797	4202	4214
Average cds length (bp)	1274	1080	1131
Largest gene (bp)	55,341	51,488	57,019
Largest cds (bp)	15,933	16,650	16,641
Number of single-exon genes	6734	11,104	5434

**Fig. 3:**
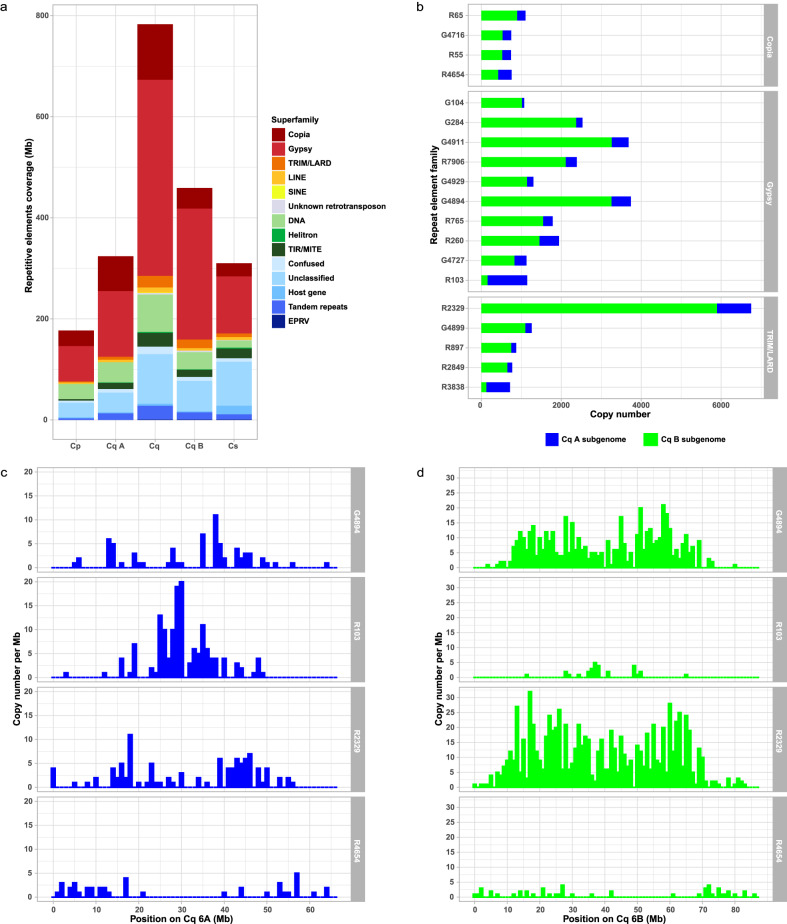
Repeat element annotation of *C. quinoa*, *C. pallidicaule* and *C. suecicum* genomes. **a** Repeat element superfamily composition in each of the three genomes and quinoa subgenomes. **b** Copy number proportions of the 19 highly repeated LTR families in each quinoa subgenome ordered by increasing logFC of enrichment between A and B subgenomes within each superfamily. **c**, **d** From the top panel to the bottom, the distribution of a B-subgenome enriched *Gypsy* family, an A subgenome enriched *Gypsy* family, a B-subgenome enriched TRIM_LARD family, and a non-subgenome-specific *Copia* family. The density of each TE family is displayed as copy number per 1-Mb windows along the Cq6A (**c**) and Cq6B (**d**) homoeolog group of chromosomes, chosen as representative of the whole genome.

A de novo annotation of the repetitive elements was performed using REPET^20,21^ for each of the QQ74-V2, *C. suecicum* V2, and *C. pallidicaul*e [20] reference assemblies. These annotations identified 782,869,030 bp (65.20%) of the quinoa genome as being repetitive, 176,230,678 bp (48.61%) for *C. pallidicaule*, and 310,035,101 (57.91%) for *C. suecicum* genomes (Supplementary Data 2). In each genome, about half of the repeat fraction (66.66% for *C. quinoa*, 57.90% for *C. pallidicaule*, 47.13% for *C. suecicum*) was comprised of Long Terminal Repeat (LTR) transposable elements, representing the most prevalent type of repeats (Fig. 3a). However, discrepancies exist in the coverage of these repeat families within each subgenome. While the A and B subgenomes account for 44.20% and 55.80% of the quinoa genome, respectively, the content of repetitive sequence in each subgenome is not proportional to their subgenome sizes, representing 323,470,441 bp (60.95%) of the A subgenome and 459,398,589 bp (68.57%) of the B subgenome (Supplementary Data 2). The overall difference (135,928,148 bp) in TE space between A and B subgenomes explains 97.61% of the size difference between the two quinoa subgenomes (139,262,428 bp) and is mostly due to LTR repeats contributing 112,037,747 bp (82.42%) of differential representation of TE families between the two subgenomes (Fig. 3a and Supplementary Data 2). Of the 438 LTR families annotated in *C. quinoa* genome (incl. 261 Gypsy, 151 Copia, and 26 TRIM-LARD elements), 72 families had a complete LTR sequence structure and were thus qualified as high-quality LTR repeat elements (Supplementary Data 3). Among these, 19 families were highly repeated (with copy numbers greater than the average LTR repeat number per family), and a majority (15 of 19) of the families are over-represented (being at list twice more abundant in copy number) in the B subgenome, whereas only two families are A subgenome-specific and one is not subgenome-specific (Fig. 3b—Supplementary Data 3). While the overall TE distribution follows a pattern of decreasing density from centromere to telomere, the distribution of the individual LTR TE families along quinoa chromosomes shows local variations, with some families being more enriched in the distal chromosomal regions while others are more enriched in the proximal regions (Fig. 3c), implying an impact of these families’ expansion on the structure of quinoa subgenomes.

Analysis of the superfamily repeat copy number and total length in each quinoa subgenome and its diploid relative indicated that the B subgenome is more dynamic than the A subgenome (Supplementary Data 4). In terms of repeat copy number, 90% of the total copy number differences (340,320) between the quinoa B subgenome and the B-genome diploid *C. suecicum* are due to copy number reductions in the quinoa B subgenome. However, despite this overall reduction in repeat copy numbers in the quinoa B subgenome, copy number expansion in a small number of superfamilies—including mainly the Gypsy, TRIM/LARD, and SINE repeat superfamilies—resulted in a substantial net increase in total repeat length (194,315,909 bp) in the quinoa B subgenome compared to *C. suecicum*. This is in agreement with recent findings in the BCD-genome hexaploid *C. formosanum*, in which it was found that a recent increase in copy number of Gypsy repeats contributed to the increased size of the B subgenome relative to the C and D subgenomes^22^. In contrast, total repeat copy numbers in the quinoa A subgenome and the A-genome diploid *C. pallidicaule* differed by only 79,399 bp, of which 99% was due to copy number expansion in the A subgenome of quinoa.

### Analysis of structural rearrangements within and between subgenomes

**Fig. 4:**
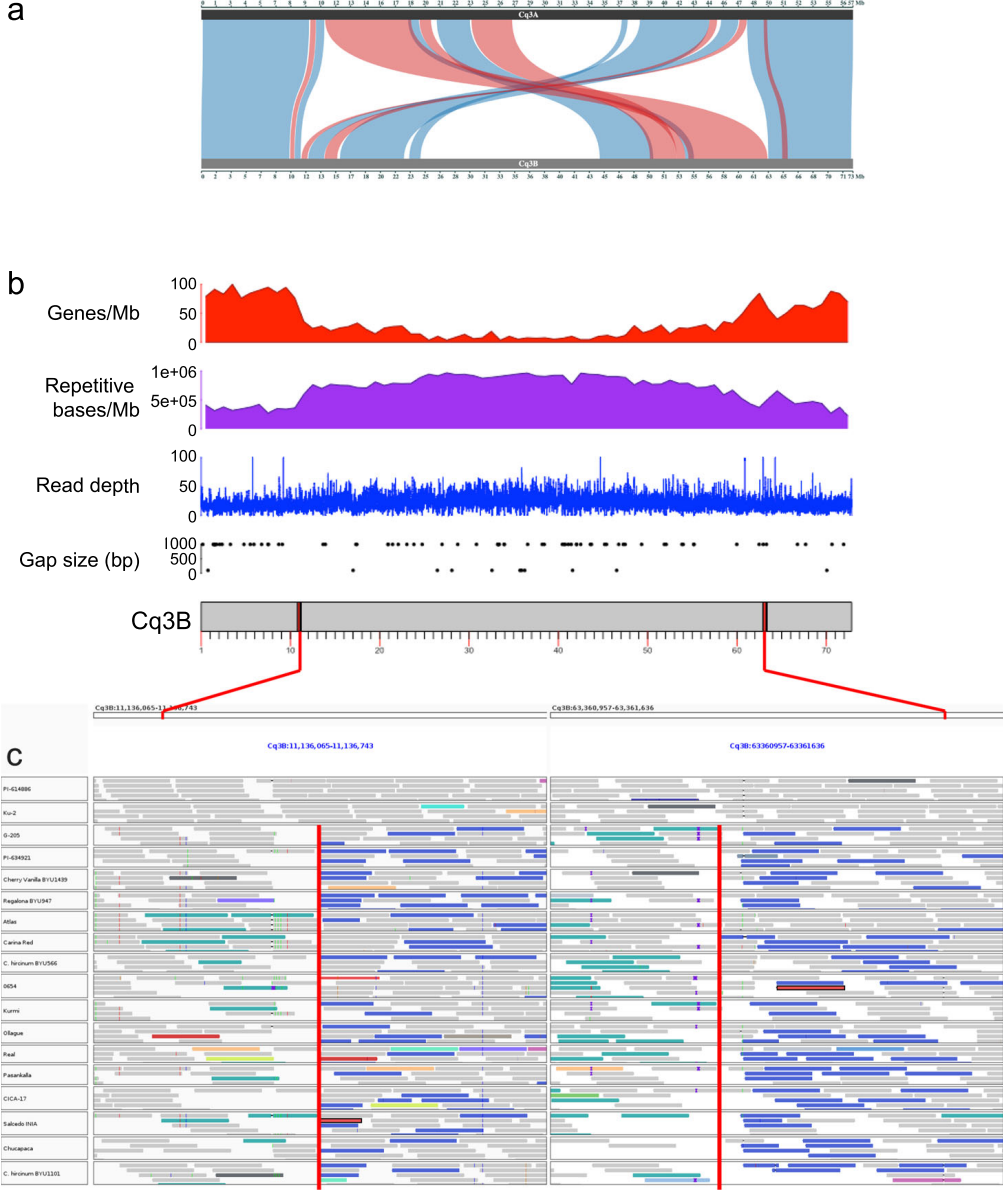
Identification and characterization of the Cq3B pericentromeric inversion. **a** Evidence for the inversion through analysis of gene synteny between the homoeologous chromosomes Cq3A and Cq3B. Synteny blocks between Cq3A and Cq3B homoeologous chromosomes are displayed by blue-colored bands between the two chromosomes, while red indicates inverted syntenic blocks. **b** Evidence for the inversion through analysis of structural features of chromosome Cq3B. The breakpoints of the Cq3B pericentromeric inversion are evidenced as red bands on the chromosome ideogram and displayed in the context of the gene (red) and TE (purple) densities in 1-Mb windows, QQ74 PacBio read mapping depth (blue), and assembly gap positions (black dots) along the Cq3B chromosome. **c** Evidence for the inversion through analysis of read mapping. Illumina reads from QQ74 mapped against QQ74 (PI 614886), 15 other quinoa accessions, and 2 *C. hircinum* genotypes are displayed under the two breakpoints of the Cq3B pericentromeric inversion, precisely identified with the dotted, vertical red line. Correctly mapped reads are shown as gray bars, and incorrectly mapped, discordant reads (such as reads whose mate maps to a different region) are displayed as dark blue and turquoise blue bars.

Analysis of the physical positions of genetic markers from the Kurmi × 0654 population mapped to the QQ74-V2 assembly revealed a break at each telomeric end of chromosome Cq3B (Supplementary Fig. 6d). The first break was situated at the proximal end between 10.8–12.4 Mb, and the second break was at the distal end between 62.9–63.8 Mb. These breaks between the physical and genetic maps also colocalize with breaks in the collinearity between Cq3A (Cq3A:10,854,916–48,035,345) and Cq3B (Cq3B: 11,108,204–63,361,684), revealing a large pericentromeric inversion of approximately 52 Mb (71.7% of the complete chromosome size) (Fig. 4a). To determine whether these breaks represent a mis-joining of contigs into scaffolds, we first investigated the presence of sequence gaps (N’s) in the pseudomolecules in the regions defined by the breakpoints (Fig. 4b). No gap was present in the proximal chromosomal region, but a gap was identified between the breakpoints in the distal region of the pseudomolecule at position Cq3B:62,938,700–62,939,700. While the genetic map was developed from a cross between two highland quinoas (Kurmi × 0654), the genome assembly was produced in QQ74, a coastal variety. We therefore hypothesized that the discrepancy between the genetic and physical maps is a result of a chromosomal rearrangement that has occurred in QQ74, a coastal quinoa variety. To validate this hypothesis and to determine the incidence of the inversion in other quinoa varieties and ecotypes, we mapped short reads from 16 previously re-sequenced quinoa accessions^6^, including eight highland and eight coastal quinoas, against the QQ74-V2 chromosome assembly. The analysis of Kurmi and 0654 read alignments in the regions of the putative breakpoints allowed us to identify the precise breakpoint positions as Cq3B:11,136,405 and Cq3B:63,361,214, as revealed by the presence of discordant reads for which one mate of the pair mapped to the Cq3B:11 Mb region and the other mate mapped to the Cq3B:63 Mb region (Fig. 4c). The identification of the precise breakpoints enabled the validation of the Cq3B pericentromeric inversion in QQ74 using PCR amplification with primer pairs flanking the left (11f–11r) and right (63f-63r) breakpoints. (Supplementary Fig. 8). These primers produced bands of the expected size in QQ74 but not in quinoa Real. Conversely, when the primer pairs were switched (11f-63f and 11r-63r), bands of the expected size were produced in Real but not in QQ74, indicating that the inversion is present in coastal QQ74 but not in highland Real. Analysis of the alignment of the 13 other quinoa accessions revealed that the inversion, which we named In(3B)(11136405::63361214), is present in one other coastal quinoa (Ku-2) but is absent from all other coastal accessions and all highland accessions (Fig. 4c).

**Fig. 5:**
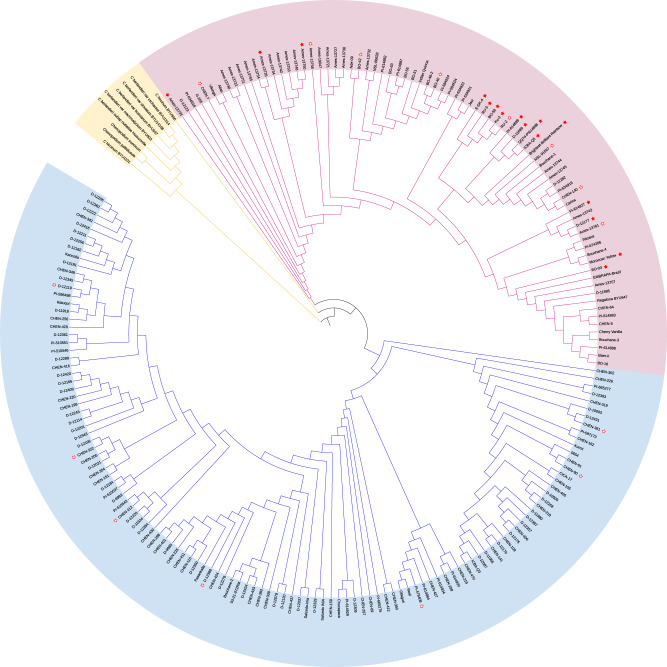
Distribution of the Cq3B pericentromeric inversion across the phylogenetic tree derived from 184 quinoa accessions representing highland and coastal germplasm. Germplasm from the related species *C. berlandieri*, *C. hircinum*, *C. pallidicaule*, and *C. suecicum* are displayed in yellow, coastal quinoa germplasm in pink, and highland quinoa germplasm in blue. Filled red stars designate quinoa accessions carrying the In(3B)(11136405::63361214) inversion, as determined by mapped reads spanning the breakpoints and the absence of discordant read pairs mapping to both breakpoint regions. Empty red stars identify accessions for which the presence of the inversion was suspected due to the absence of read mapping under the breakpoint, but not supported by discordant read mapping.

In order to better understand the implications of this rearrangement for recombination and gene flow within and between coastal and highland germplasm, we investigated the presence of this Cq3B pericentromeric inversion in a set of 184 previously re-sequenced quinoa accessions^23^ representing greater genetic diversity. Because QQ74 contains the Cq3B inversion, accessions for which sequencing reads map discordantly (defined as read pairs for which one mate maps to the Cq3B:11 Mb region, and the other maps to the Cq3B:63 Mb region) in the QQ74-V2 assembly likely do not contain the inversion. A total of 155 (84%) accessions (47 and 108 are coastal and highland accessions, respectively) contained one or more discordant reads at both sites and were therefore determined to be unlikely to carry the Cq3B inversion (Table 4). In addition to QQ74 and Ku-2, 14 accessions, all of which were coastal ecotypes, contained reads spanning the breakpoints and were therefore determined to likely carry the Cq3B inversion. Fifteen accessions, comprised of eight coastal and seven highland ecotypes, lacked reads mapping to the breakpoints and were therefore determined to be inconclusive (Supplementary Fig. 9, Supplementary Data 5). We generated a phylogenetic tree of all accessions to clarify the phylogenetic distribution of accessions containing the inversion. Although 11 of the 14 coastal accessions carrying the In(3B)(11136405::63361214) inversion are clustered together, the three other coastal accessions containing the inversion are distributed among the coastal germplasm, providing no evidence for genetic drift of quinoa accessions carrying this rearrangement (Fig. 5).

**Table 4:** Distribution of the Cq3B pericentromeric inversion In(3B)(11136405::63361214) in a panel of 184 quinoa accessions.

	Coastal	Highland
Total accessions	69	115
Contain the Cq3B inversion (%)	14 (20.3)	0 (0)
Do not contain the Cq3B inversion (%)	47 (68.1)	108 (93.9)
Inconclusive (%)	8 (11.6)	7 (6.1)

To investigate additional potential rearrangements between quinoa subgenomes, we performed a synteny analysis between quinoa subgenomes as well as with their diploid relatives *C. pallidicaule* and *C. suecicum*. For this, we performed peptide alignment and synteny analyses between 53,042 quinoa, 21,193 *C. pallidicaule*, 18,105 *C. suecicum*, and 20,459 *B. vulgaris* sequences from the contigs anchored to the nine haploid quinoa chromosome sets using MCScanX. With a minimum MATCH_SIZE of eight as the minimum number of genes to define a collinearity block, 77,376 genes (70.15%) out of the 112,799 complete genes were collinear across all species (Supplementary Data 6). Among these, 29,742 genes (56.07%) are collinear between the quinoa subgenomes (Fig. 2a), while 30,255 (64.14%) and 24,110 (53.37%) are collinear between the A and B subgenomes of quinoa and their respective diploid genome relatives *C. pallidicaule* (Fig. 2b) and C. *suecicum* (Fig. 2c). While these results suggest that the quinoa A subgenome is more highly conserved than the B subgenome, these values could also reflect the more fragmented nature of the reference genome assembly of *C. suecicum* compared with *C. pallidicaule*, which could adversely affect synteny with their respective quinoa subgenomes. Six major (greater than 1 Mb in length and containing greater than 100 genes) chromosomal rearrangements were identified between quinoa subgenomes and their diploid relatives, as well as seven minor rearrangements within and between subgenomes (Supplementary Data 7). Breaks in collinearity between homoeologous pairs of chromosomes and lack of synteny between rearranged chromosomes and their diploid relatives enabled the identification of two reciprocal translocations between Cq1B and Cq2B and between Cq7B and Cq9B, and five translocations between Cq2A and Cq7A, Cq2B and Cq4A, Cq4A and Cq2B, Cq9A and Cq5A, and Cq9A and Cq8A (Fig. 2, Supplementary Data 5 and 6, Supplementary Fig. 10). Duplicated synteny blocks allowed the identification of two intra-chromosomal segmental duplications on Cq1A and Cq2B, and one inter-chromosomal duplication between Cq4B and Cq2A (Fig. 2, Supplementary Data 5 and 6, Supplementary Fig. 10). A synteny comparison between QQ74-V2 and a newly reported chromosome-scale assembly of the A-genome diploid *C. watsonii*^24^ confirmed the presence of the Cq1B-Cq2B, Cq7B-Cq9B, and Cq2B-Cq4A reciprocal translocations. Finally, patterns of diploid *C. pallidicaule* and *C. suecicum* read mapping against the quinoa subgenomes allowed the identification of an additional reciprocal translocation between the homoeologous chromosomes Cq6A and Cq6B (Supplementary Data 5-8, Supplementary Fig. 10). Altogether, 43,067,200 bp of the quinoa genome is rearranged, 86.31% of which is between chromosomes of the same subgenome (Supplementary Data 8). The rearrangements account for 2,865 (11.03%) genes and 3,336,041 bp (0.63%) of the A subgenome of quinoa, and 4,003 (14.79%) genes and 39,731,159 bp (5.93%) for the B subgenome (Supplementary Data 8), and all occurred post-polyploidization, supporting the previous results indicating that the B subgenome of quinoa is more dynamic than the A subgenome (Supplementary Data 4 and 8).

## Discussion

Higher-quality genome assemblies are becoming increasingly necessary to support the complete discovery of allelic variation contributing to complex traits, especially in the context of large, highly repetitive and polyploid genomes such as plant genomes^25^. Since the release of the first chromosome-scale reference assembly of quinoa genome in 2017^6^, we observed an exponential increase of work published on the characterization of the genetic variation underlying important quinoa domestication traits such as flowering time^26–29^, drought^30–33^, salinity^7,34–37^, and heat tolerances^31,38–41^. This reveals the continuous interest in improving the quality of the quinoa reference genome and increase the availability of genomic resources such as gene and TE annotations, as well as whole-genome resequencing of the genetic diversity. A key objective of this study was to improve the quinoa reference genome assembly both in terms of sequence contiguity and assembly accuracy. Compared to the former V1 PB+BN+Chi reference assembly^6^, the QQ74-V2 assembly showed a 17-fold increase in scaffold N50 length and added a total of 17.3 Mb new sequences and 10,616 newly annotated genes to the sequences anchored to the 18 chromosomes. This new genome represents an improvement for the quinoa reference in terms of composition and structure of the assembled pseudomolecules, which was demonstrated by a higher gene collinearity between homoeologous chromosomes and a better agreement of chromosome lengths with cytogenetics observations^9^.

Another important goal of this study was to improve the reference genomes and annotations of C. *pallidicaule* and *C. suecicum* diploids species in order to provide resources to support evolutionary and structural analyses of A and B subgenomes of quinoa. In this paper, we release an updated genome reference assembly and gene annotation of *C. suecicum* together with high-quality de novo TE annotations of all three quinoa, *C. pallidicaule* and *C. suecicum* genomes. These resources provide the substrate for better characterization of the structure and dynamic of quinoa subgenomes through the identification of eight translocations, three segmental duplications, and one large pericentromeric inversion which, collectively, involve all homoeologous groups of quinoa chromosomes. As genomic reshuffling is known to be a source of genetic novelty, the identification of these chromosomal rearrangements provides the framework for investigating the potential impact of quinoa genome structure on adaptation and speciation through the formation of novel genes^42^, modification of expression patterns^43^, and creation of linkage between previously unlinked genes^44^.

The refined TE annotation for all three genomes represents an additional asset to investigate subgenomes dynamics and its potential impact on the structure and function of the quinoa genome. Our results of the repeat family dynamic between the quinoa subgenomes and their diploid relatives confirm the findings by Schiavinato et al. (2021) that the quinoa allotetraploid genome has been shaped by biased fractionation^45^. However, our results, which focused on the repetitive fraction of the genome, lead to different conclusions about the dynamic of the quinoa subgenomes. Our data support the conclusion that the biased fractionation of quinoa subgenomes resulted from a higher dynamic and expansion of the repeat fraction of the B-subgenome rather than by the loss of sequence from the A subgenome, as was suggested by the whole-genome analysis (including repetitive and genic sequences together) of mapping diploid reads against the quinoa genome performed by Schiavinato et al. (2021). We believe that the results of higher loss or remodeling of the quinoa A subgenome found by Schiavinato et al. reflect the large evolutionary distance of *C. pallidicaule* as a diploid relative to the quinoa A subgenome, rather than its subgenome dynamic.

Finally, it will be interesting to investigate how translocations and inversions contribute to the evolution and structure of quinoa diversity. In the present study, we evidenced a large pericentromeric inversion on chromosome Cq3B, which we found to be shared exclusively among coastal genotypes within the 184 accessions investigated. The current data do not allow for a conclusion on genetic drift of populations carrying the rearranged allele, and therefore suggest that the inversion is large enough to allow for recombination through looping of the chromosome^46^. Further work is needed to understand the potential disruption of meiotic pairing or the suppression of recombination in rearranged chromosomal regions^47^. Future work should also be conducted in order to fully appreciate the representation of such chromosomal rearrangements in the quinoa diversity and potential impact on quinoa germplasm evolution.

## Methods

### Input assemblies and scaffolding with in vivo Hi-C

We previously produced the quinoa QQ74 V1 assembly using PacBio CLR reads scaffolded with a combination of Bionano Irys optical maps, Dovetail Chicago in vitro Hi-C, and a genetic linkage map. To produce the quinoa QQ74 V2 assembly, in vivo Hi-C scaffolding was performed with three input assemblies based on data from the V1 assembly: the Pacbio (PB) and PacBio+BioNano (PB + BN) assemblies were previously reported^6^, and the PacBio+Chicago in vitro Hi-C assembly (PB+Chi) was newly produced by scaffolding the PB assembly using previously reported Chicago Hi-C data^6^ and the HiRise assembler by Dovetail Genomics. Scaffolding of these three input assemblies was performed with in vivo Hi-C data using the Proximo assembler by Phase Genomics as previously described^48^, using 174,994,553 pairs of Illumina sequencing reads. Juicebox^49,50^ was used to manually correct scaffolding errors.

For *C. suecicum*, in vitro Chicago and in vivo Hi-C scaffolding was performed by Dovetail Genomics with the previously reported ALLPATHS assembly^6^.

### Gap-filling and polishing

The PB+Chi Hi-C assembly was determined to be the most correct of the three quinoa Hi-C assemblies (see Results). Gap-filling of this assembly was performed with PBJelly^51^ implemented in pbsuite v15.8.24, using the same PacBio reads used to produce the assembly, and using the “--capturedOnly”, “--spanOnly”, and “-m 2” arguments. The gap-filled assembly was polished using Pilon^52^ v1.22 and the Illumina sequencing data previously reported^6^. Specific computational parameters for these and other methods described herein can be found in the Supplementary Methods. This final assembly, produced by gap-filling and polishing the Hi-C-scaffolded PB+Chi assembly, is referred to as the QQ74-V2 assembly.

The *C. suecicum* Hi-C assembly was also gap-filled and polished as described above. Gap-filling was performed using newly produced PacBio sequencing reads; polishing was performed using previously reported Illumina reads^6^. This final assembly is referred to as the *C. suecicum* V2 assembly.

### Assessing assembly completeness

Completeness of the new QQ74-V2 assembly, the original V1 PB+BN+Chi+linkage assembly^6^, and the *C. suecicum* V2 assembly was assessed with four different scores. BUSCO^53^ scores were obtained with BUSCO v5.0.0 using the embryophyta_odb10.2019-11-20 dataset. LTR Assembly Index (LAI) scores were generated using LTR_retriever^54^ v2.8.7 using recommended parameters “-minlenltr 100 -maxlenltr 7000 -mintsd 4 -maxtsd 6 -motif TGCA -motifmis 1 -similar 85 -vic 10 -seed 20 -seqids yes” for the ltrharvest step. K-mer completeness scores were computed using Merqury^16^ v1.3 and a default k-mer size of 19 to build the k-mer database. Consensus k-mer QV score was measured through exact (Meryl) method. Finally, mapping rate of Illumina sequences back to the assembly contigs were obtained through read mapping using bowtie2 v2.5.1 in end-to-end mode with default ‘--sensitive’ parameter. Mapping rates were output from BAM files using the ‘flagstats’ function of samtools v1.7.

### Gene and repeat annotation

Repetitive sequences were identified in the QQ74-V2 and *C. suecicum* V2 assemblies using RepeatModeler^55^ v1.0.11, and repeats were classified using RepeatMasker^56^ v4.1.2 with RepBase database version 20160829. Genes were annotated using MAKER^57^ v3.01-beta. For quinoa, ab initio gene prediction was performed with AUGUSTUS^58^ v3.5.0, using gene models and hidden Markov gene models from *Arabidopsis thaliana*. Evidence for quinoa gene models was provided by coding sequences (CDS) and peptide sequences from the *Beta vulgaris* BeetSet-2 genome annotation;^59^ a transcriptome derived from previously reported quinoa RNA-seq data^6^, generated by mapping reads to the genome assembly using HISAT2^60^ v2.1 and assembling into transcripts using StringTie^61^ v1.3.4; exon hints derived from previously reported quinoa Iso-seq data^6^, generated by mapping reads to the genome assembly using minimap2^62^ v2.12 and creating a bam2hints file using bam2hints in AUGUSTUS; and the uniprot_sprot database (downloaded May 2019).

Ab initio gene prediction in *C. suecicum* was also performed with AUGUSTUS, using hidden Markov gene models from *A. thaliana* and BUSCO gene models from *C. berlandieri* and *A. thaliana*. Evidence for *C. suecicum* gene models was provided by transcript and peptide sequences from quinoa, a previously reported transcriptome from *C. suecicum*^6^, and the uniprot_sprot database (downloaded May 2019).

### Transposable elements annotation using REPET

We ran a de novo repeat detection using the TEdenovo v2.4 pipeline from the REPET package v2.4^20^ on the *C. suecicum*, *C. pallidicaule* and *C. quinoa* assemblies. From each assembly, a genomic subset ranging 340–370 Mbp was generated and used as input to launch TEdenovo v2.4 with parameters set to consider repetitive elements with at least 5 copies. The resulting libraries of consensus sequences were then compared to their respective genomic subsets using the TEannot^21^ v2.4 pipeline to keep only those that are found at least once as full-length copy. Each filtered library was then used as digital probe for whole-genome annotation with TEannot v2.4. Each library of consensus sequences was classified using PASTEC^63^ followed by semi-manual curation.

### Assessing gene annotations quality and completeness

Completeness of the new QQ74-V2 annotation, the original V1 PB+BN+Chi+linkage annotation^6^, and the *C. suecicum* V2 annotation were assessed with BUSCO v4.0.4^53^ in ‘proteins’ mode using the embryophyta_odb10 dataset (Creation date: 2017-12-01, number of species: 40, number of BUSCOs: 1375). Annotation statistics were performed using AGAT v0.4.0^64^. The comparisons of the gene models from the original V1 PB+BN+Chi+linkage annotation and those from the new QQ74-V2 annotation through the measure of intron-chains conservation were performed using GFFCompare v0.11.2^65^.

### Assessing gene synteny and collinearity between the different assemblies

To ensure comparable analyses of gene synteny and collinearity among the various quinoa assemblies, genes from the previously reported genome annotation of the original quinoa assembly^6^ were transferred to the new quinoa assemblies using the default parameters of flo^66^. For analyses involving *B. vulgaris*, version EL10.2^67^ (available at genomevolution.org, genome ID 57232) was used. Pairs of homologous genes within quinoa and between quinoa, *B. vulgaris, C. suecicum*, and *C. pallidicaule* were identified using BLASTp v2.9.0 with the “-num_descriptions 5”, “-num_alignments 5”, and “-evalue 1e-10” arguments. Clusters of collinear gene pairs were identified using the default parameters of MCScanX^68^ and visualized with VGSC2^69^.

### Comparing genetic and physical marker positions

We previously mapped RNA-seq reads from 45 F3 progeny of Kurmi × 0654 to the PB+BN+Chi assembly and generated a linkage map from SNPs identified in reads of each individual in the population^6^. We identified the physical positions of these SNP markers in each new Hi-C assembly by extracting 500 bp of sequence (250 bp upstream and 250 bp downstream) flanking each marker in the PB+BN+Chi assembly and then performing a BLASTn v2.9.0 search of these sequences in each Hi-C assembly. We selected only the single top hit for each query sequence and plotted the physical position of these top hits in each assembly relative to the mapped genetic position of each marker.

### Repeat elements coverage and enrichment analyses

The REPET curated annotation was used to investigate the representation (in base pairs and copy number) of repeat elements in the allotetraploid *C. quinoa* genome and subgenomes, and the *C. pallidicaule* (A) and *C. suecicum* (B) diploid genomes. Differential repeat copy number between each quinoa subgenome and its diploid relative was computed at the superfamily level in order to assess the repeat dynamics (expansion or retraction of a given repeat family) following polyploidization in the allotetraploid genome. Further to this, LTR repeat enrichment and distribution were evaluated in the quinoa subgenomes by selecting complete high-quality families annotated as full-length elements by TEannot v2.4 during the REPET annotation process. Subgenome enrichment or specificity was assessed as LTR families with a minimum LOG2 differential copy number variation of one (or a minimum fold-change of two) between the two subgenomes. The copy number density of four LTR families along the quinoa chromosome Cq6B was computed in 1-Mb windows using bedtools v2.19.1^70^, and the distribution along the chromosome was plotted using ggplot2^71^ using R^72^ v4.3.1^73^.

### Identification of chromosomal rearrangements in the quinoa genome

With the aim to identify major chromosomal rearrangements within and between quinoa subgenomes, we first performed a refined synteny analysis by extracting clusters of collinear gene pairs from the above synteny analysis using the parameter ‘MATCH_SIZE 8’ of MCScanX^68^ as the minimum number of homologous gene pairs required to define a collinearity block. Synteny between quinoa subgenomes and between quinoa and their diploid A and B diploid was then plotted at the genome level using Circos v0.69-9^74^, or at the chromosome level using SynVisio (synvisio.github.io). Gene and repeat elements densities in 1-Mb windows were computed using bedtools^70^ v2.29.1 from the gene and repeat GFF annotation files, respectively, for the QQ74_V2, *C. pallidicaule*, and *C. suecicum* genomes. Translocations were then detected through breaks in collinearity between homoeologous pairs of chromosomes and lack of synteny between the rearranged chromosomes and their diploid relative genomes. Segmental duplications were identified as duplicated blocks of genes with conserved gene order within or between chromosomes. Reciprocal translocations between homoeologous chromosomes were identified by the inverted pattern of diploid genome read mapping against quinoa subgenomes. For this, Illumina short-reads whole-genome sequencing data previously produced^6^ for *C. pallidicaule* (SRR4425239) and *C. suecicum* (SRR4425238) were aligned to QQ74-V2 using Bowtie v2.3.4.1^17^ in *--end-to-end* mode using the *--sensitive* mapping parameter. The output BAM files were then sorted using samtools v1.7^75^ and directly used for read mapping visualization using IGV v2.12.3^76^ or as an input to mosdepth v0.3.1^77^ to calculate average mapping rates in 1-Mb windows along QQ74-V2 chromosomes with a minimum mapping quality threshold *-q 20*. Inverted patterns of diploid read mapping were identified as regions where the average mapping of the expected diploid relative (*C. pallidicaule* for the A subgenome, and *C. suecicum* for the B-subgenome of quinoa) was inferior or equal to half of the overall mapping rate on its related subgenome, while the average mapping of the alternative diploid relative was equal or superior to half the overall mapping rate on its related subgenome. The mapping of PacBio long reads sequencing of QQ74 (SRR4279763) against QQ72-V2 using minimap2 v2.15 with default parameters was used to visually check the coverage under the breakpoints of each chromosomal rearrangement using IGV (v2.12.3) to exclude the possibility of a mis-assembly.

### Identification of Cq3B pericentromeric inversion

In order to precisely identify the breakpoints of the Cq3B pericentromeric inversion, we mapped previously produced^6^ Illumina resequencing reads of Kurmi and 0654, the two parental accessions of the mapping population from which the genetic map was developed, as well as QQ74 and the 15 other quinoa resequencing lines against QQ74-V2 using bwa-mem v0.7.17^78^ with default parameters except for mapping quality threshold set to *‘-q 20*’. The breakpoints of the inversion were identified through visualization of read mapping on IGV in the immediate (<50 kb distance) vicinity of the synteny breaks between Cq3A and Cq3B, within the intervals of breaks between physical and genetic markers’ alignment, through the detection of read mapping coverage sharp interruption at the positions 11,136,405 and 63,361,684, and presence of discordant reads with one mate mapping the first breakpoint and the other mapping the second breakpoint. In order to screen the presence of the inversion in the wider representation of the geographical and genetic quinoa diversity, we collected Illumina short-read resequencing data available for 310 quinoa accessions^23^ and mapped them against QQ74-V2 using bwa-mem (v0.7.17) with default parameters and mapping quality threshold set to *‘-q 20*’. Only accessions resulting in more than five-times coverage of the 1.45 Gb quinoa genome size after mapping were retained for further analyses, resulting in a set of 184 quinoas. Discordant reads with a mapping insert size comprised between 52,223,700 and 52,225,600 bp were counted in the mapping intervals Cq3B:11136100-11137000 and Cq3B:63360700–63361700 encompassing both breakpoint regions. We then scored the presence of the QQ74-type of Cq3B inversion as accessions with one or more discordant read at both sites, and manually checked the mapping under both breakpoints for the accessions presenting no discordant reads in order to conclude for the ‘absence’ of the QQ74-type of Cq3B inversion in the cases where at least one read was mapping across the breakpoints, and ‘not conclusive’ in the case where the read mapping coverage around the breakpoints was not sufficient to conclude on the presence or absence of the inversion.

### PCR validation of the Cq3B pericentromeric inversion

DNA was extracted from leaves of quinoa Real and QQ74 plants using the Qiagen DNeasy Plant Mini Kit. PCR was performed using the ThermoFisher DreamTaq Hot Start PCR Mastermix, following the manufacturer’s recommended protocol. Sequences of the 11 f, 11r, 63 f, and 63r primers were ACCCCAACTCCGATACAGTG, CGTTTTCGTCATGTGGATTG, ACGATCCGGTGTCAAAACTC, and GCTTCAATTGGAGACCCAAA, respectively. Annealing temperatures of 50° C and 56° C were used for the 11f-11r and 63f-63r primer pairs, respectively. An annealing temperature of 55° C was used for the 11f-63f and 11r-63r primer pairs. PCR products were separated on a 1% agarose gel at 100 V for 40 minutes.

### Distribution of the Cq3B pericentromeric inversion

In order to infer the spread of the Cq3B pericentromeric inversion across the quinoa genetic diversity, we mapped all 25 resequencing genotypes^6^ (including 16 quinoas, 5 *C. berlandieri*, 2 *C. hircinum* and the diploids *C. pallidicaule* and *C. suecicum*) and 184 quinoa accessions^23^ described above against QQ74-V2 and produced a phylogenetic tree from the SNP variants identified following the previously described method^23^. For this, reads of all 209 accessions were mapped against QQ74-V2 using BWA-MEM v-0.7.17 with default parameters except for mapping quality threshold set to *‘-q 20*’ and then BAM files were sorted and indexed using samtools v1.8. Duplicated reads were marked, and read groups were assigned using the Picard tools (http://broadinstitute.github.io/picard/). Variants were identified with GATK v4.1.8.0^79^ using the “--emitRefConfidence” function of the HaplotypeCaller algorithm and “—heterozygosity” value set to 0.005 to call SNPs for each accession. Individual g.vcf files for each sample were then compressed and indexed with tabix v-0.2.6^60^ and combined into chromosome g.vcf using GenomicsDBImport function of GATK. Joint genotyping was then performed for each chromosome using the function GenotypeGVCFs of GATK. SNPs were further filtered to retain biallelic variants only, with a minimum read depth of 5 under each genotype called (--minDP 5), a maximum of 20% missing data across all samples (--max-missing 0.8), and a minimum allele frequency of 1% (--maf 0.01). This filtering yielded a total of 493,271 high-quality SNPs across 209 samples which were then converted to PHYLIP format using vcf2phylip v-1.5^80^. The phylogenetic tree was built following the previously described method^23^ using IQ-TREE v-2.1.4-beta^81^ with the GTR + F + R8 model for tree construction (GTR: General time-reversible, F: Empirical base frequencies, R8: FreeRate model) and 1000 bootstrap replicates. The phylogenetic tree was then visualized using the Interactive Tree Of Life tool (https://itol.embl.de/)^82^.

### Statistics and reproducibility

All new DNA sequencing data was produced from multiple leaves of a single plant for each sequencing application. No experiments required replication. Transposable elements subgenome enrichment was assessed on highly repeated LTR families (with an overall family copy number in the *C. quinoa* genome greater than the average LTR copy number of *n* = 698). Differential representation of each LTR family between the two subgenomes was determined by LOG2 differential copy number variation of one (or a minimum fold-change of two) between the two subgenomes. The prevalence of the Cq3B pericentromeric inversion chromosomal rearrangement was studied across *n* = 184 genotypes from 8 different countries. The phylogenetic tree supporting the analysis of the distribution of the Cq3B pericentromeric inversion chromosomal rearrangement was constructed using 1000 bootstrap replicates with IQ-TREE.

### Reporting summary

Further information on research design is available in the Nature Portfolio Reporting Summary linked to this article.

### Supplementary information

Supplementary Information

Supplementary Data 1–8

Reporting summary

Description of Additional Supplementary Files

## Data Availability

Specific codes and parameters used for computational methods can be found in the Supplementary Methods and can be used without restrictions.

## Data availability

Sequence data that support the findings of this study have been deposited in GenBank under BioProject accession code PRJNA940147, accession numbers SRR23675102—SRR23675108. Genome assemblies and annotations can be accessed from Dryad (https://datadryad.org/) using doi:10.5061/dryad.kwh70rz70 and from CoGe (https://genomevolution.org/coge/) using the Genome id60716 for QQ74_V2 and id52047 for *C. suecicum* V2. The numerical source data for graphs presented in Fig. 3 can be found in Supplementary Data 2 and 3. All other data are available from the corresponding author upon request.
