# Supplementary material for: A chromosome-scale assembly of the quinoa genome provides insights into the structure and dynamics of its subgenomes
Source: Commun Biol. 2023 Dec 13;6:1263. doi: 10.1038/s42003-023-05613-4 (PMC10719370; doi:10.1038/s42003-023-05613-4)
Supplement: Supplementary file 1 — Supplementary Information [file 42003_2023_5613_MOESM1_ESM.pdf]

Supplementary Figures

Supplementary Figure 1. Overview of the data used to produce the original quinoa QQ74 V1 assembly, the intermediate V2 assemblies using *in vivo* Hi-C, and the final V2 assembly.

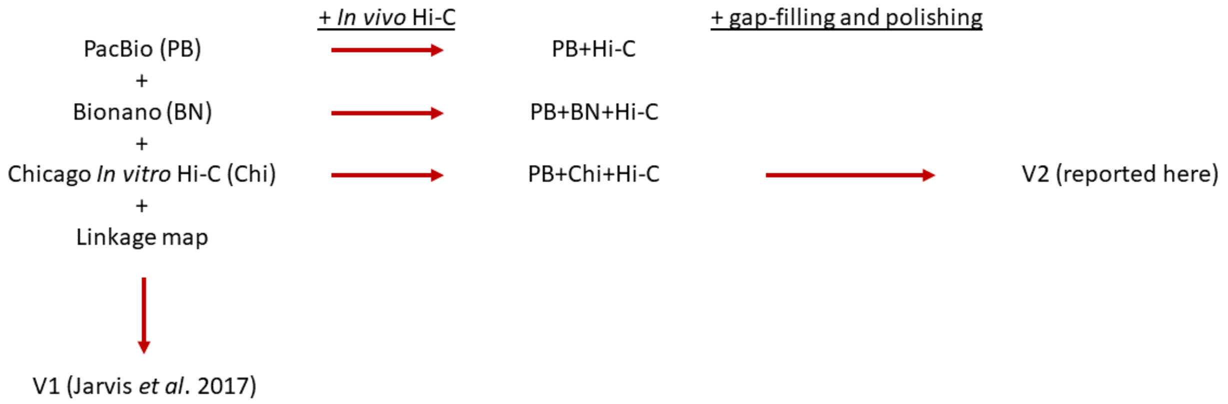

Supplementary Figure 2. Lengths of the 30 largest ordered contigs/scaffolds from the input and Hi-C output assemblies. a PB. **b** PB+BN. **c** PB+Chi.

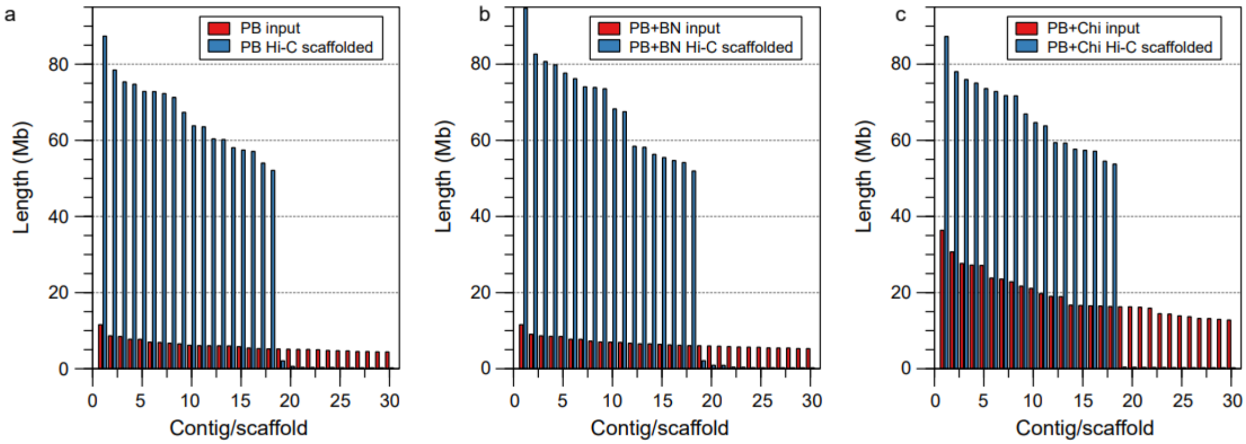

Supplementary Figure 3. Chromosomal subgenome assignments in the three Hi-C assemblies. a-c Assignments were made based on gene collinearity (red dots) between the V1 assembly and the PB (**a**), PB+BN (**b**), and PB+Chi (**c**) Hi-C assemblies. Labels for the *y*-axis in (**a**) are the same in (**b**) and (**c**).

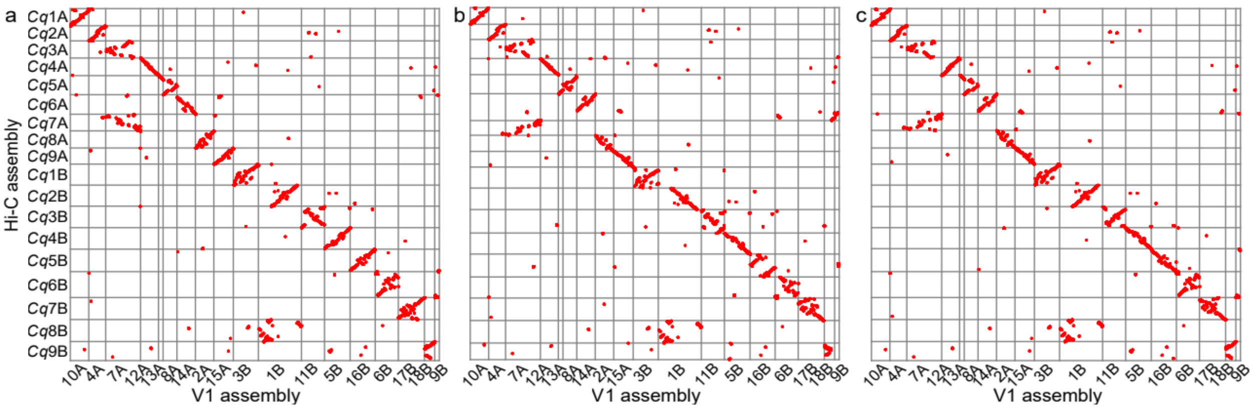

Supplementary Figure 4. Collinear relationship between *B. vulgaris* chromosomes and chromosomes of the A and B subgenomes of the Hi-C assemblies. a PB. **b** PB+BN. **c** PB+Chi.

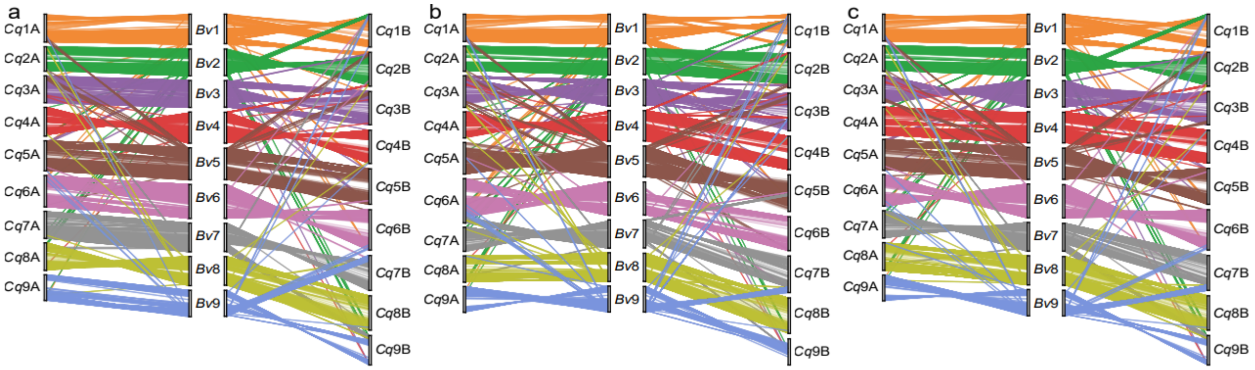

Supplementary Figure 5. Collinear gene pairs between subgenomes of the Hi-C assemblies. a PB. **b** PB+BN. **c** PB+Chi.

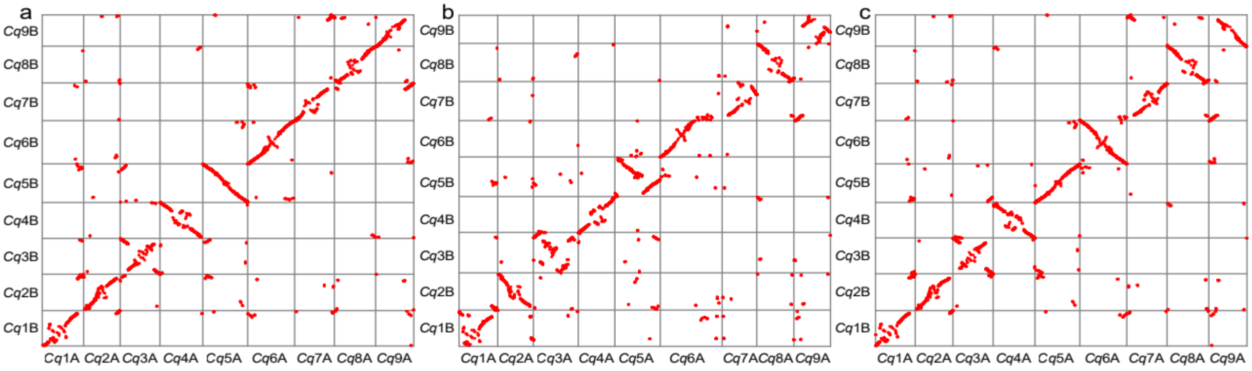

Supplementary Figure 6. Comparison of physical and genetic positions of mapped SNP markers in the Hi-C assemblies. a PB. **b** PB+BN. **c** PB+Chi. **d** QQ74-V2.

a

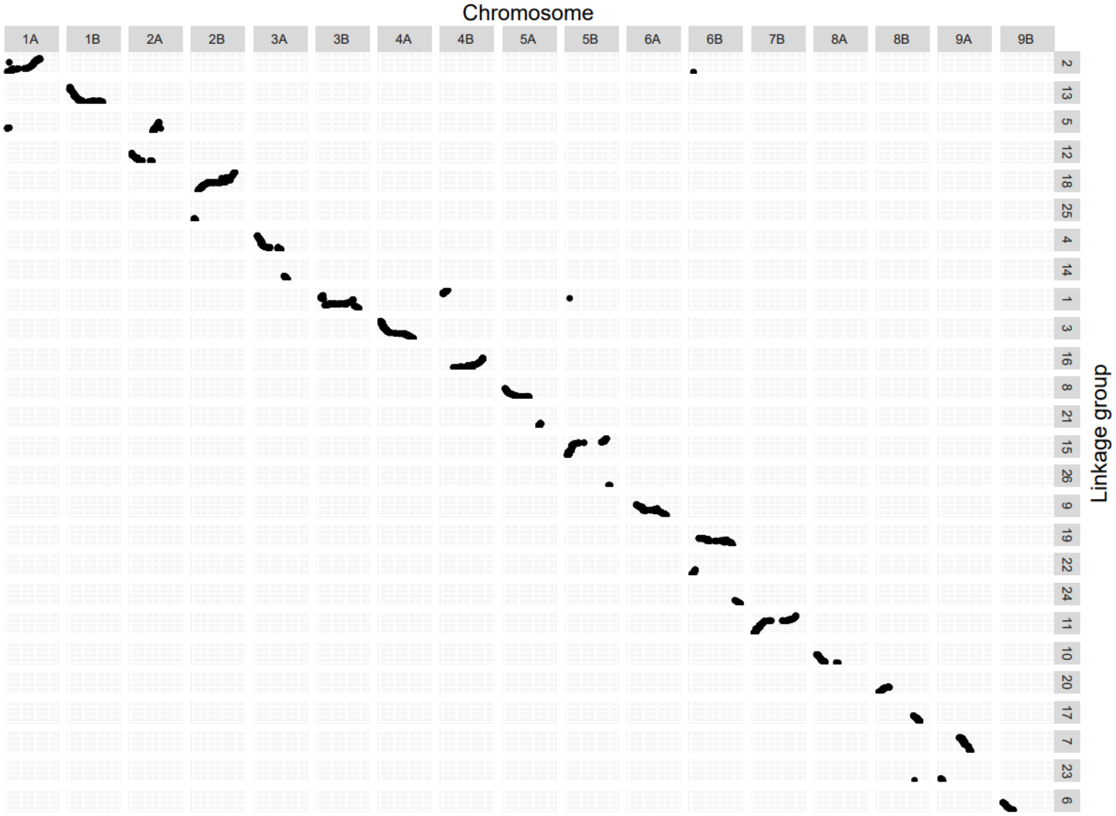

b

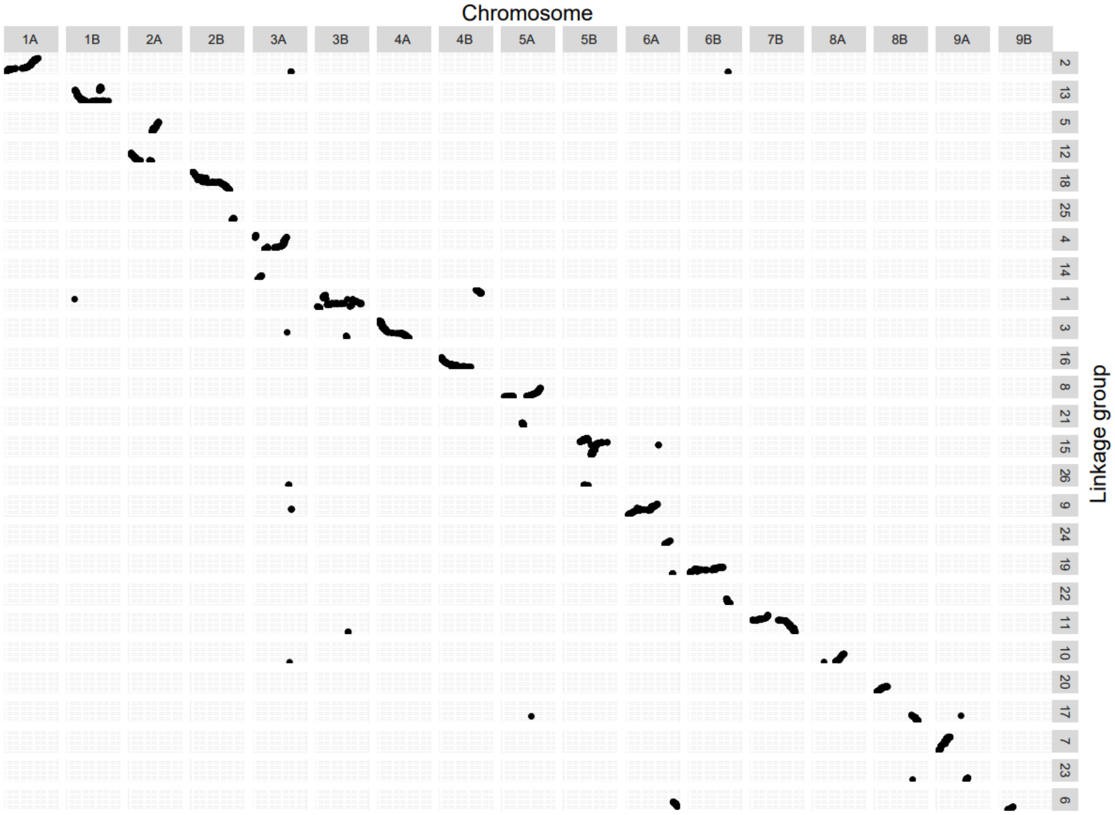

c

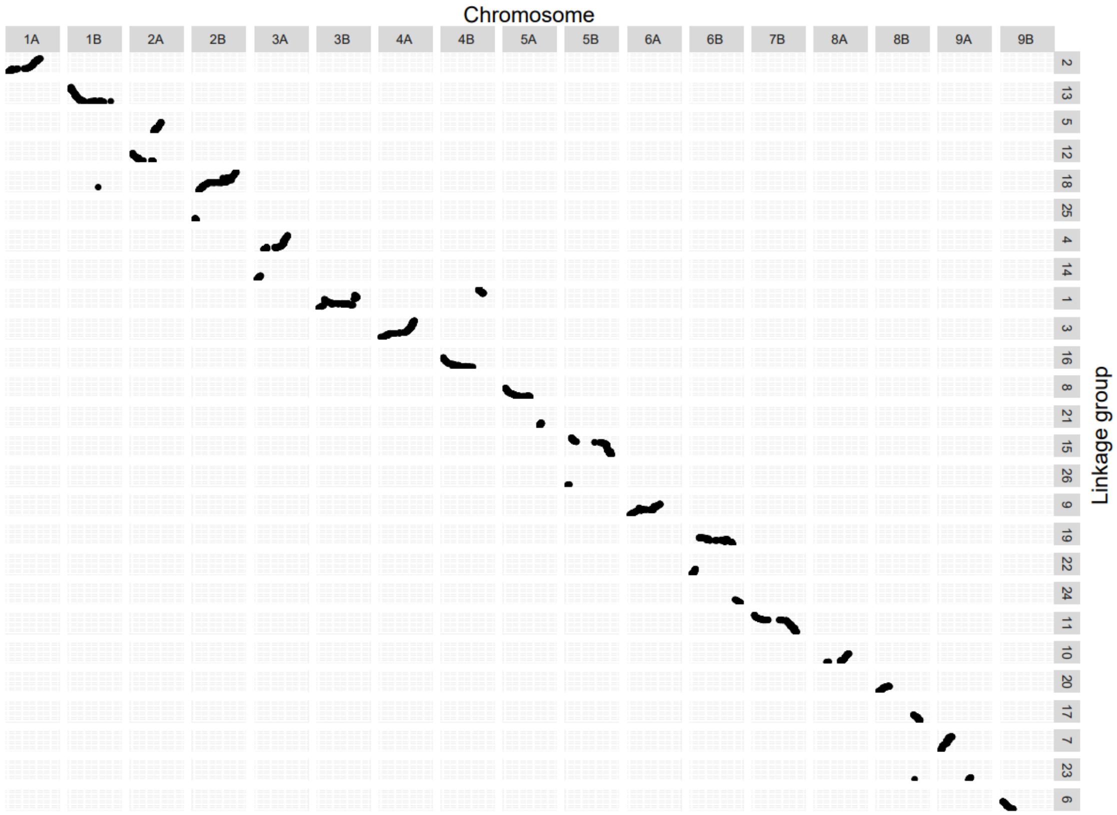
Chromosome
1A
1B
2A
2B
3A
3B
4A
4B
5A
5B
6A
6B
7B
8A
8B
9A
9B
2
13
5
12
18
25
4
14
1
3
16
8
21
15
26
9
19
22
24
11
10
20
17
7
23
6
Linkage group

d

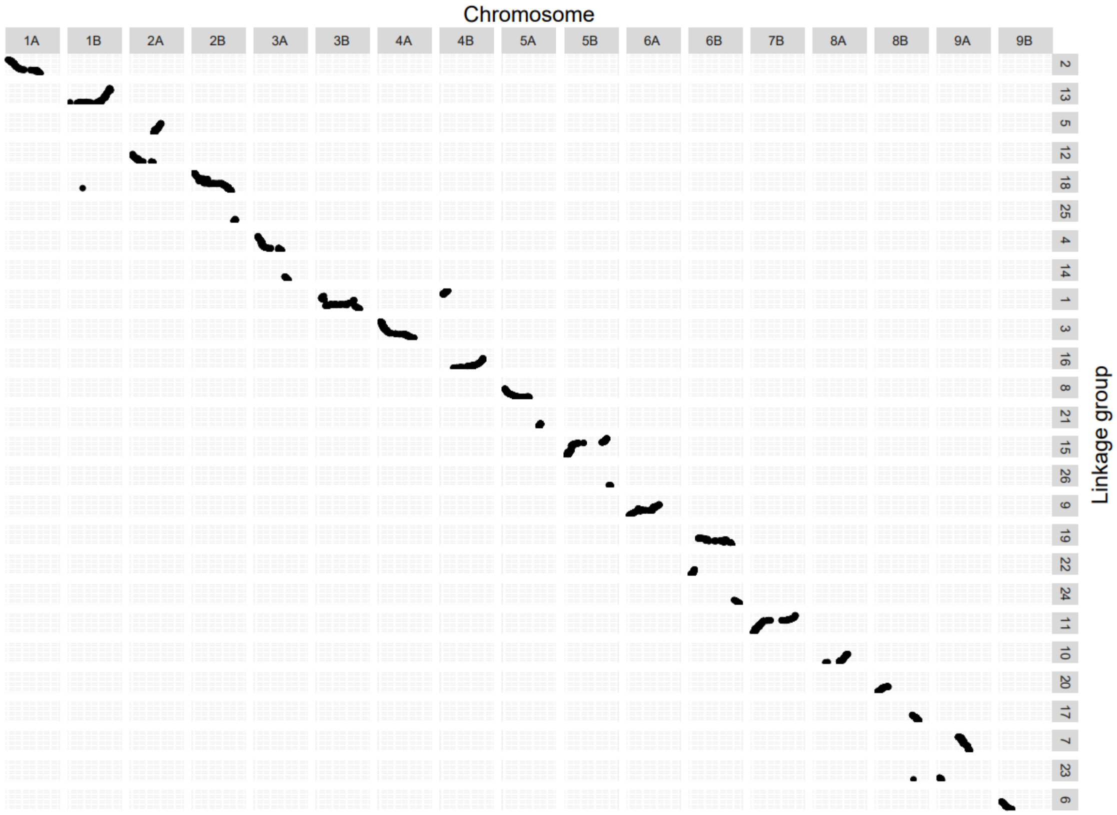
Chromosome
1A
1B
2A
2B
3A
3B
4A
4B
5A
5B
6A
6B
7B
8A
8B
9A
9B
2
13
5
12
18
25
4
14
1
3
16
8
21
15
26
9
19
22
24
11
10
20
17
7
23
6
Linkage group

Supplementary Figure 7. Saturation curves of AED scores for V1, QQ74-V2, and *C_suecicum* gene annotations.

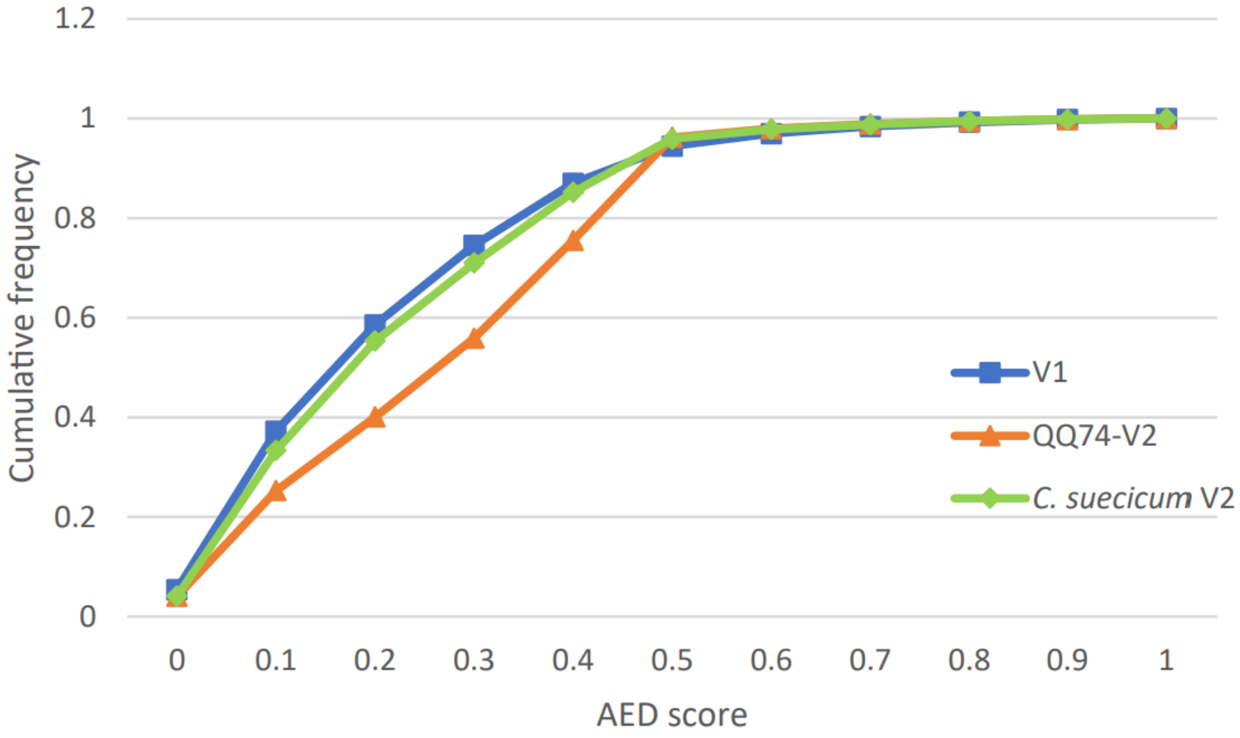

Supplementary Figure 8. Validation of the Cq3B pericentromeric inversion through PCR. Lane 1, GeneRuler 1 kb Plus DNA Ladder (sizes shown in bp). Lanes 2, 3, 6, and 7, QQ74 template DNA. Lanes 4, 5, 8, and 9, Real template DNA. Lanes 2 and 4, amplification with the 11f-11r primer pair, which is predicted to amplify a ~1,000-bp product in QQ74 but not in Real. Lanes 3 and 5, amplification with the 63f-63r primer pair, which is predicted to amplify a ~1,300-bp product in QQ74 but not in Real. Lanes 6 and 8, amplification with the 11f-63f primer pair, which is predicted to amplify a ~1,000-bp product in Real but not in QQ74. Lanes 7 and 9, amplification with the 11r-63r primer pair, which is predicted to amplify a ~1,300-bp product in Real but not in QQ74.

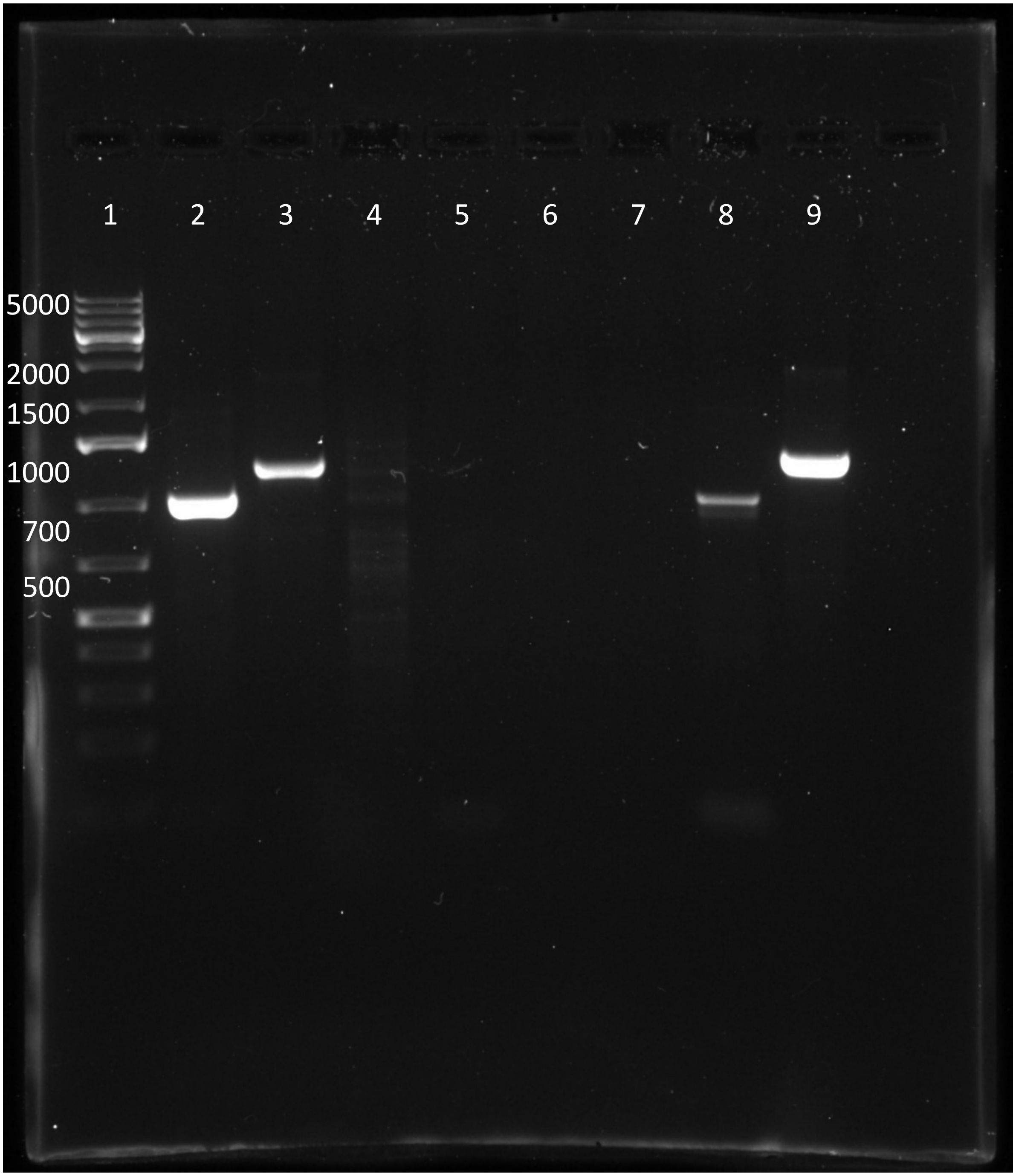

Supplementary Figure 9. Examples of scoring the presence of the Cq3B pericentromeric inversion based on read mapping of the 184 re-sequenced quinoa accessions. The top two panels (QQ74 [PI 614886] and Ku-2) represent read mapping in genotypes containing the Cq3B inversion. The middle two panels (BO-40 and Chen-322) represent genotypes for which the presence of the inversion cannot reliable be determined based on read mapping. The bottom two panels (0654 and Kurmi) represent read mapping in genotypes that do not appear to contain the inversion.

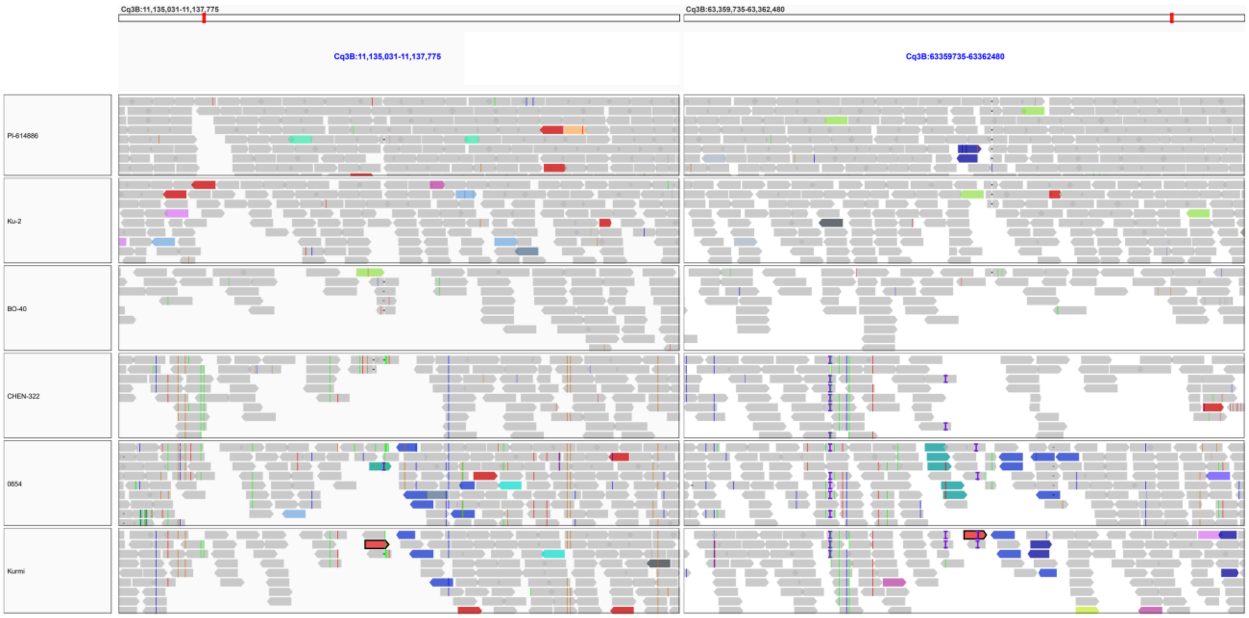

Supplementary Figure 10. Chromosomal rearrangements between and within quinoa subgenomes. Synteny blocks between homoeologous chromosomes are displayed by blue colored bands between the two chromosomes, and red color indicates inverted syntenic blocks. **a-c** Reciprocal translocations between Cq1B and Cq2B (**a**), Cq6A and Cq6B (**b**), and Cq7B and Cq9B (**c**). **d-f** Segmental duplications within Cq1A (**d**) and Cq2B (**e**), and between Cq2A and Cq4B (**f**). **g-k** Translocations from Cq2A to Cq7A (**g**), Cq2B to Cq4A (**h**), Cq4A to Cq2B (**i**), Cq9A to Cq5A (**j**), and Cq9A to Cq8A (**k**).

a

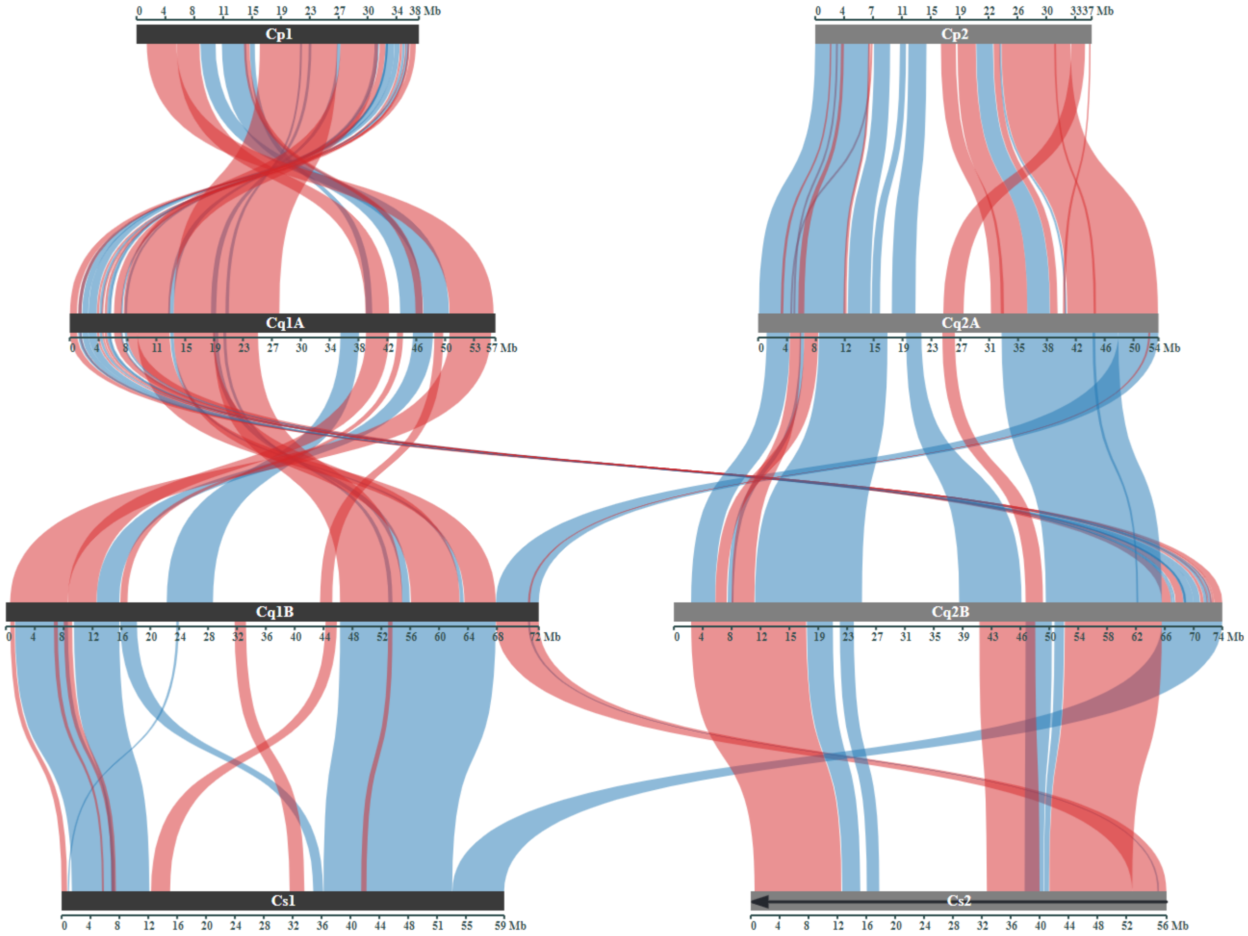

b

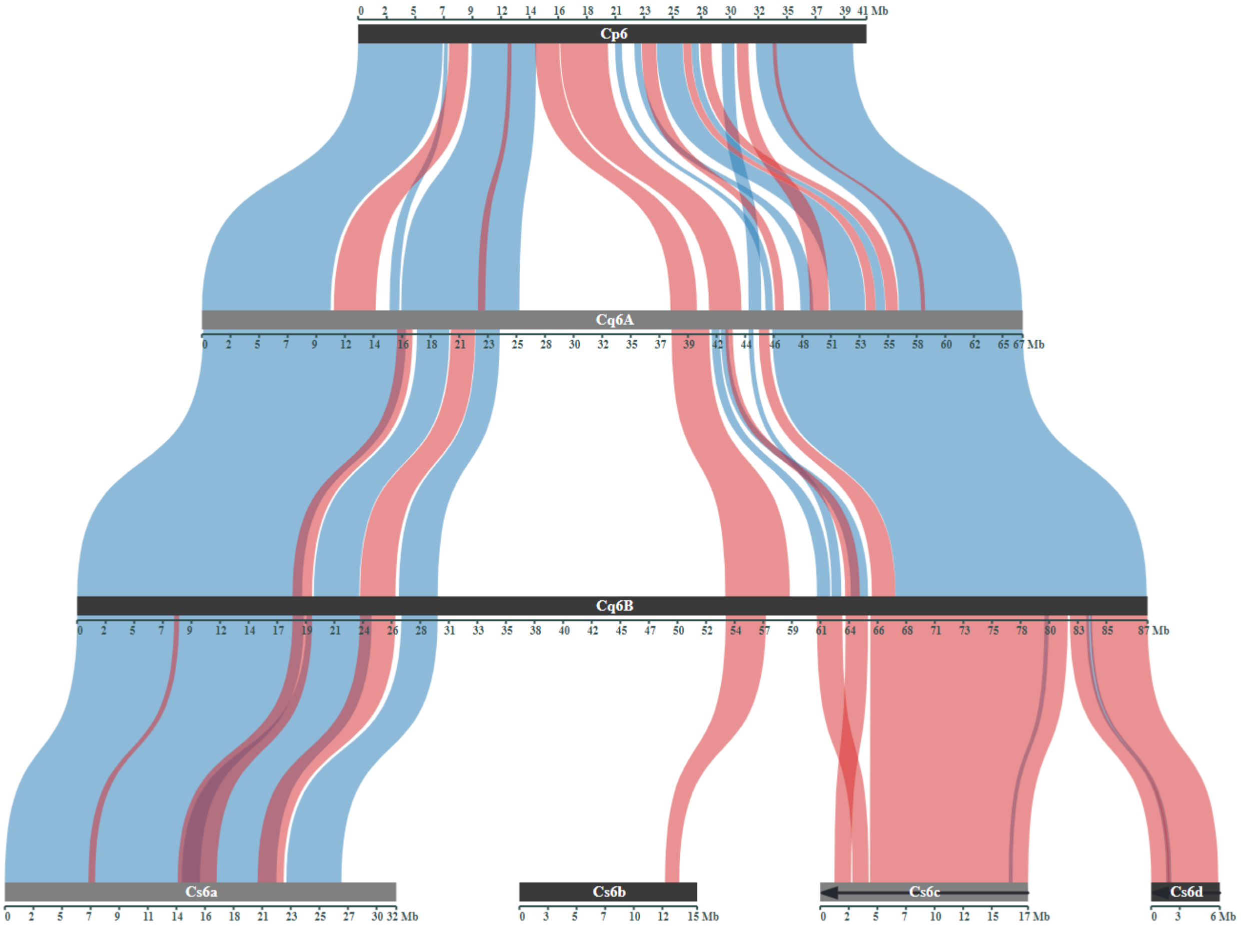

c

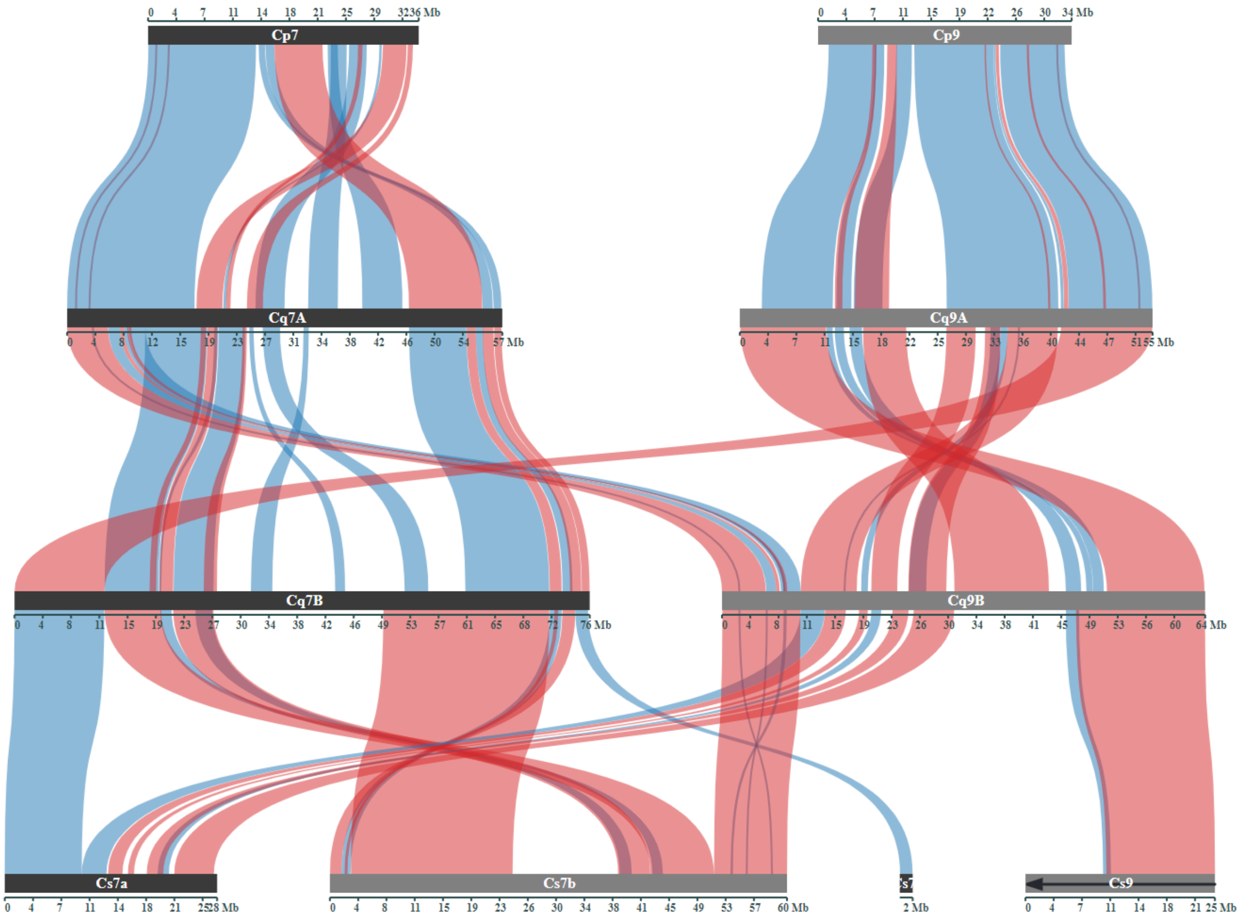

d

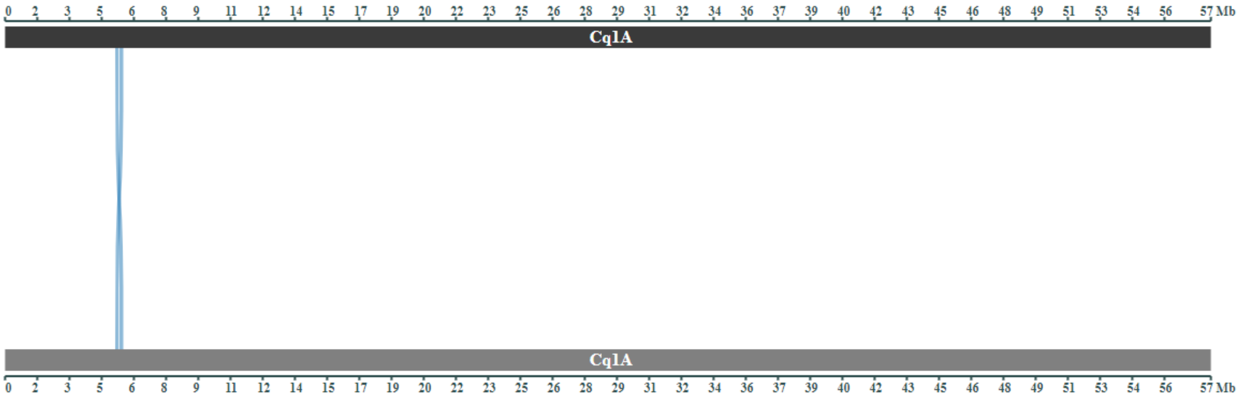

e

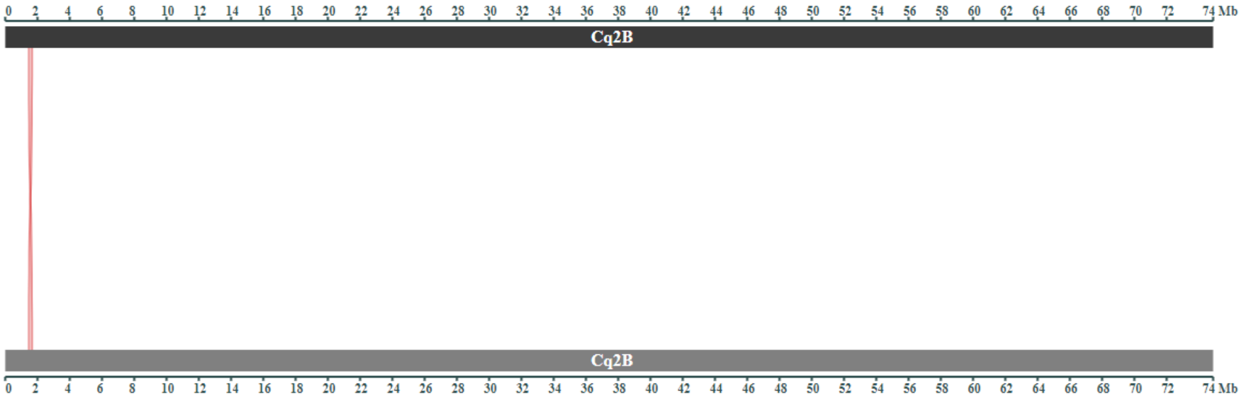

f

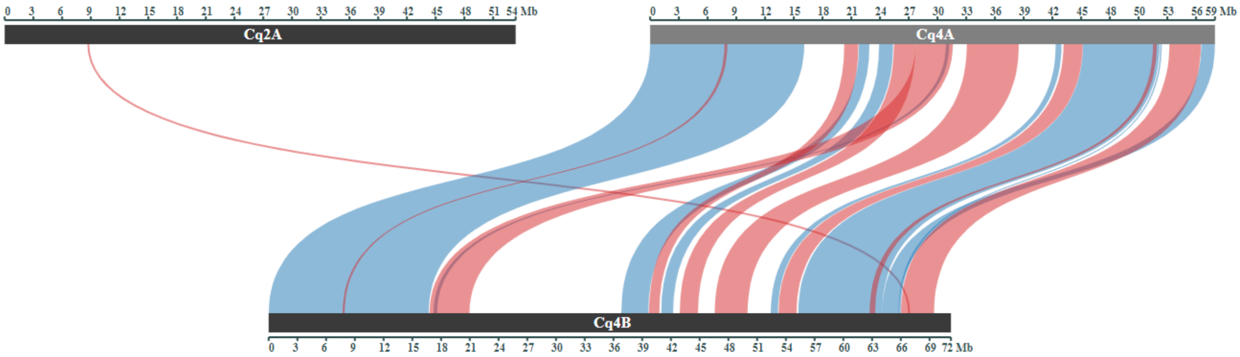

g

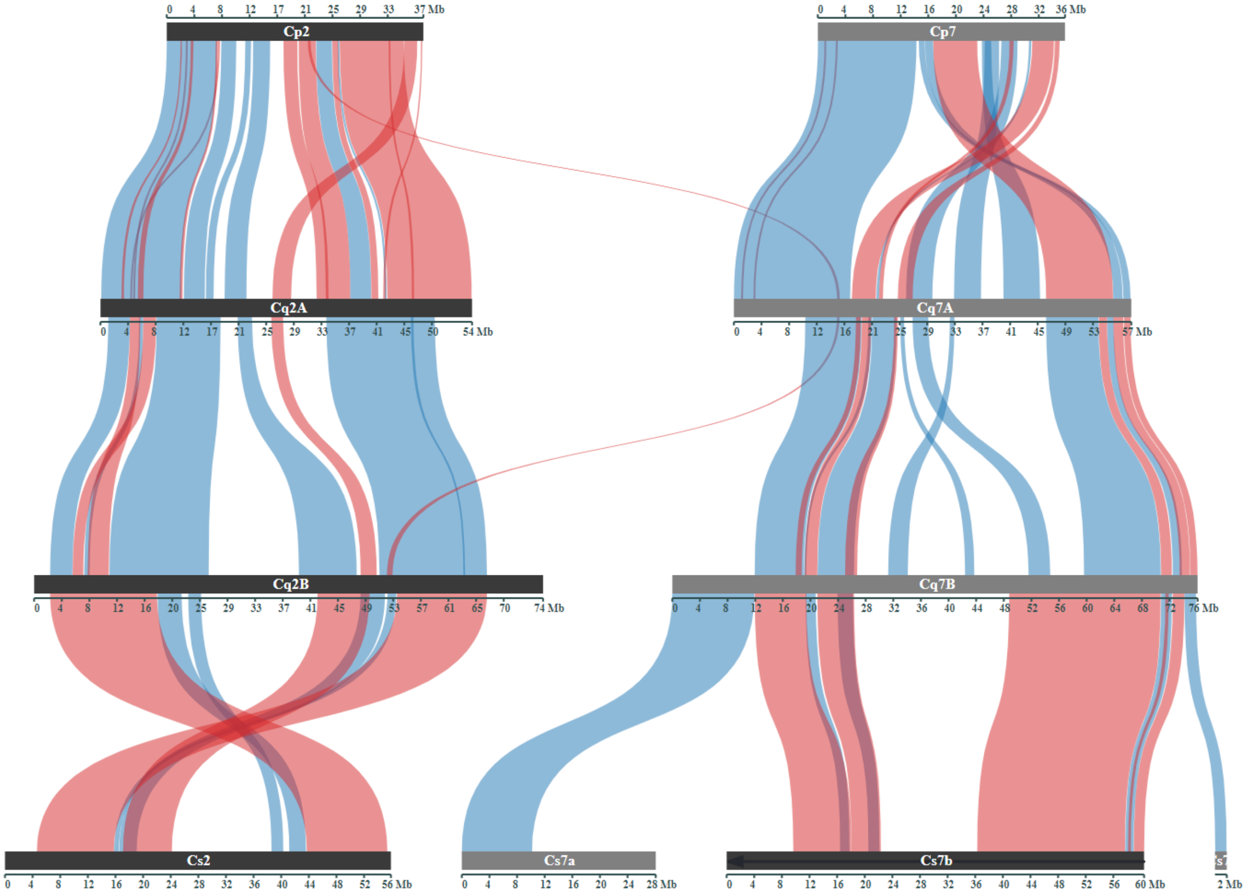
Cp2
Cp7
Cq2A
Cq7A
Cq2B
Cq7B
Cs2
Cs7a
Cs7b
0 4 8 12 17 21 25 29 33 37 Mb
0 4 8 12 16 20 24 28 32 36 Mb
0 4 8 12 17 21 25 29 33 37 41 45 50 54 Mb
0 4 8 12 16 21 25 29 33 37 41 45 49 53 57 Mb
0 4 8 12 16 20 25 29 33 37 41 45 49 53 57 61 65 70 74 Mb
0 4 8 12 16 20 24 28 32 36 40 44 48 52 56 60 64 68 72 76 Mb
0 4 8 12 16 20 24 28 32 36 40 44 48 52 56 Mb
0 4 8 12 16 20 24 28 Mb
0 4 8 12 16 20 24 28 32 36 40 44 48 52 56 60 Mb
2 Mb

h

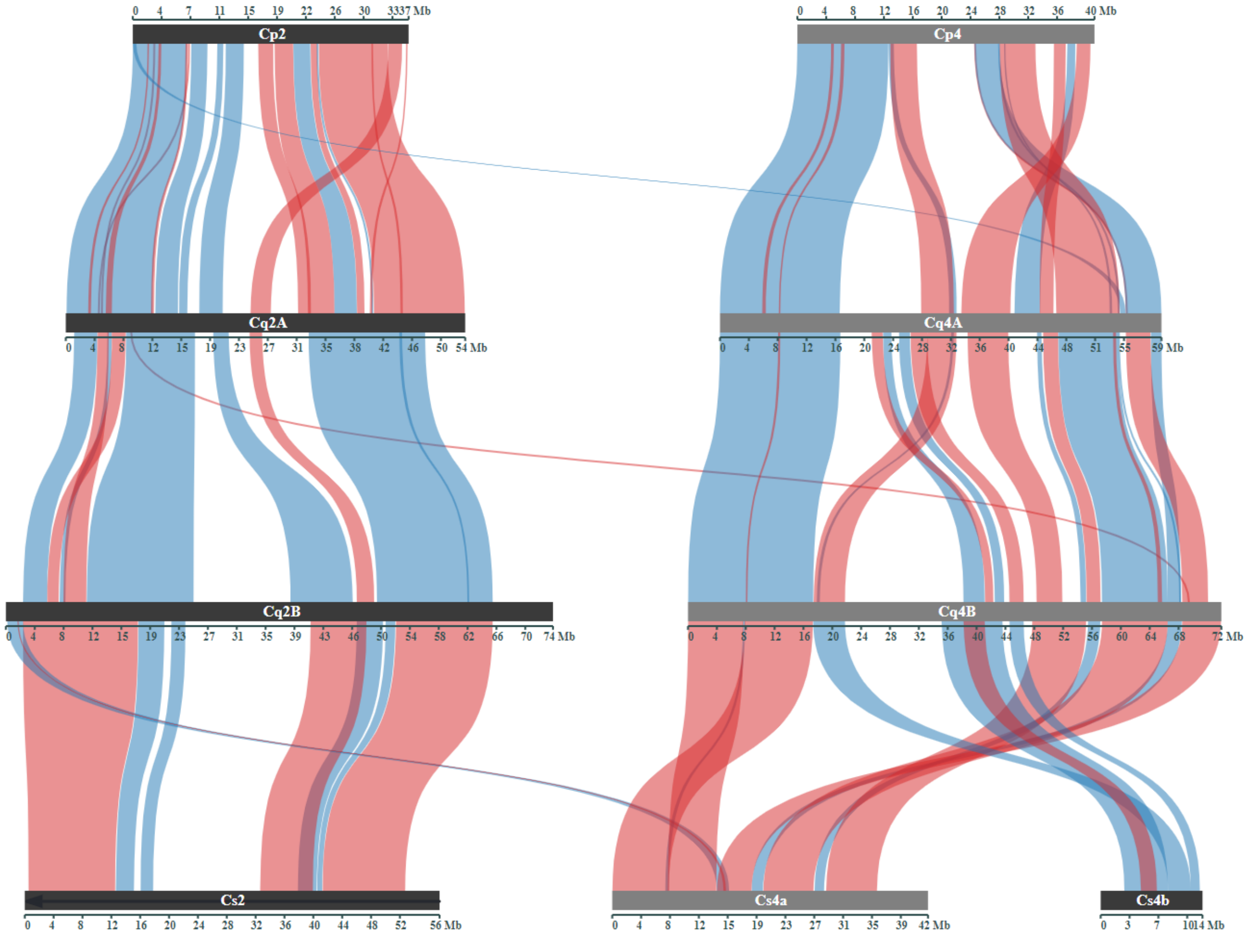

i

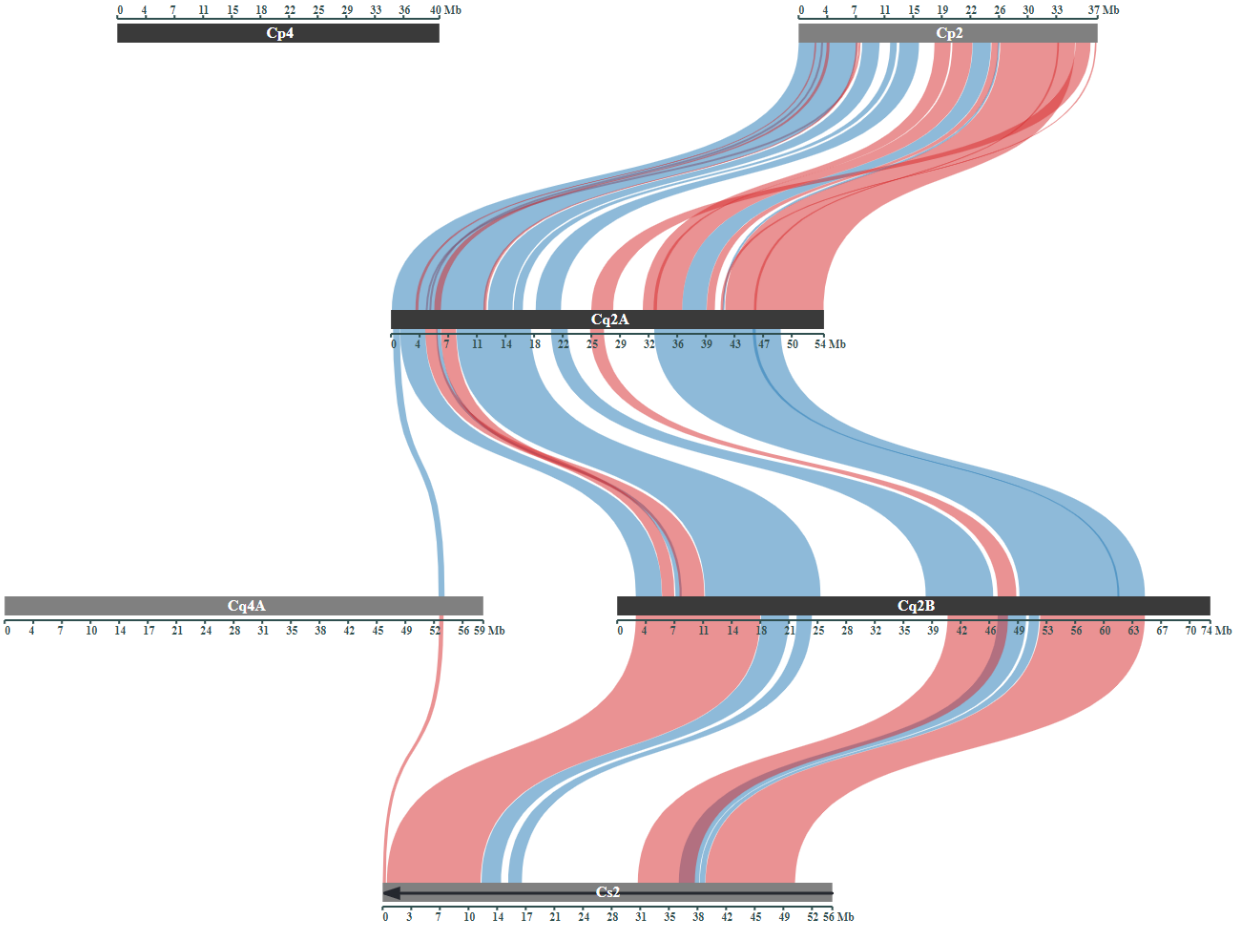

j

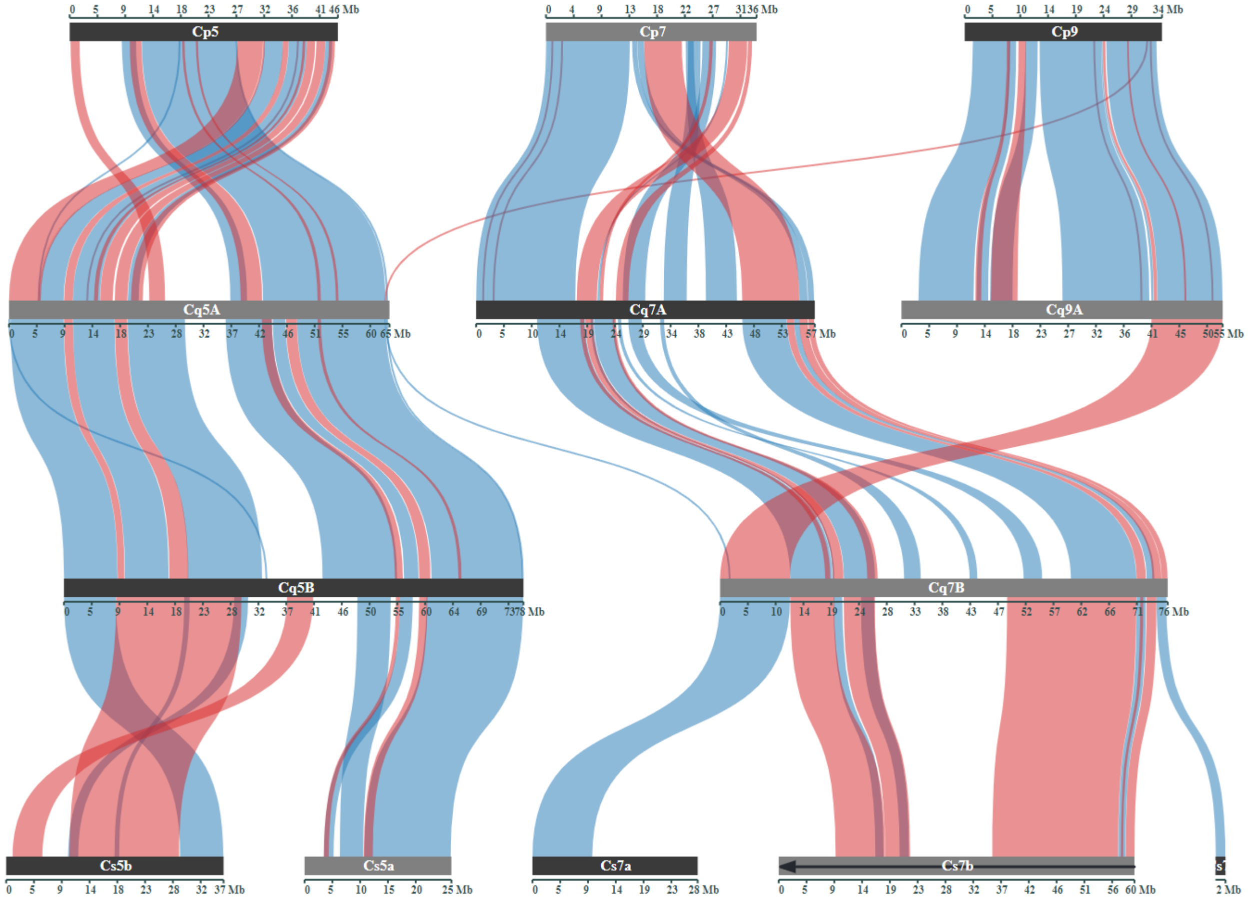

k

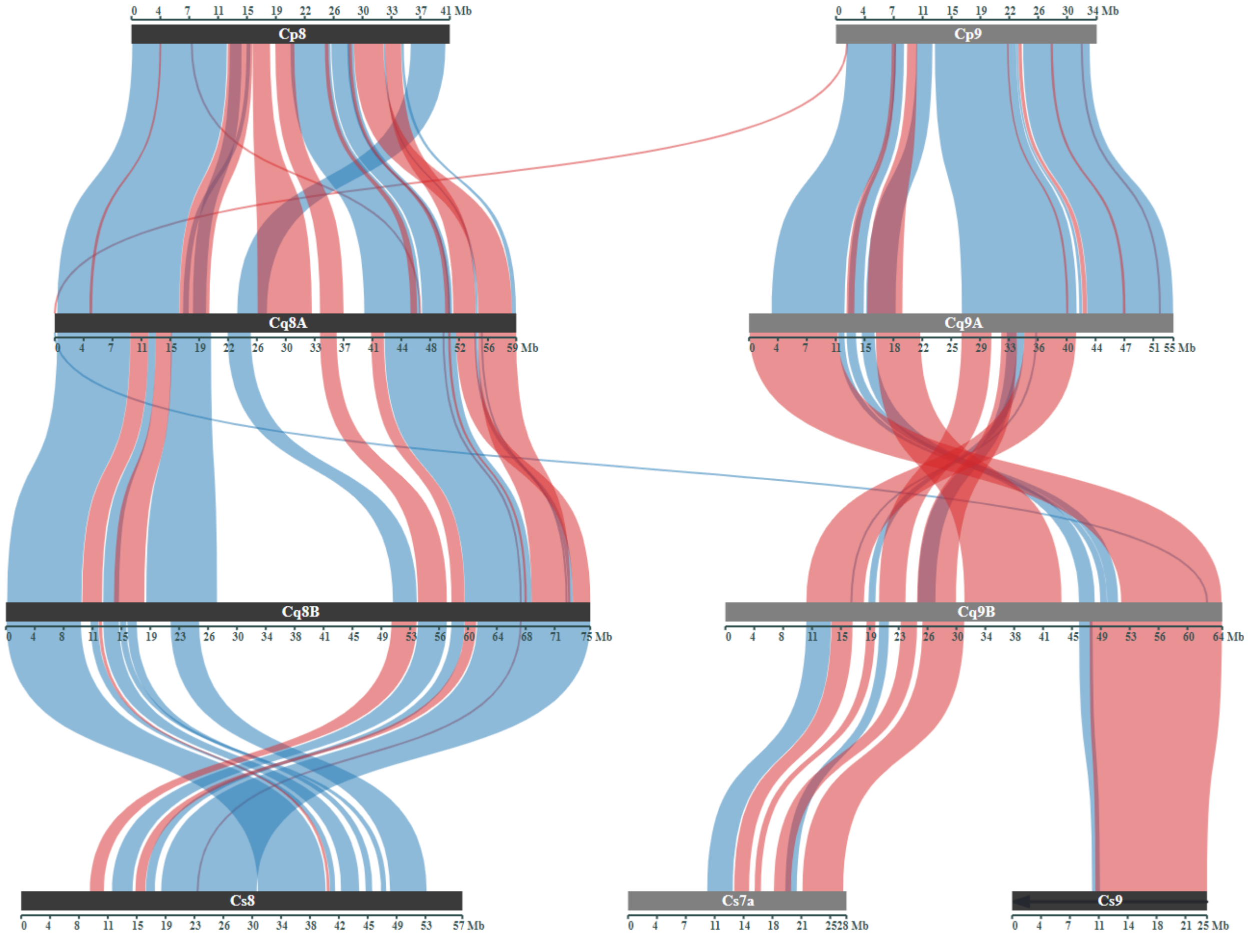

Supplementary Tables

Supplementary Table 1. Statistics of three quinoa QQ74 assemblies before and after scaffolding with in vivo Hi-C.

	PB		PB+BN		PB+Chi	
	Input	Hi-C scaffolded	Input	Hi-C scaffolded	Input	Hi-C scaffolded
Total assembly size (Gb)	1.325	1.325	1.395	1.386	1.326	1.326
Number of contigs/scaffolds	4,232	3,208	4,014	3,370	3,127	2,942
Longest contig/scaffold (Mb)	11.561	87.369	11.561	94.715	36.366	87.287
Contig/scaffold N50 (Mb)	1.663	67.345	2.451	73.555	11.753	66.923
Contig/scaffold L50	216	9	177	9	38	9
Contig/scaffold N90 (Mb)	0.139	52.098	0.157	0.188	0.348	53.755
Contig/scaffold L90	1,065	18	800	41	180	18
% in 18 largest contigs/scaffolds	9.3	90.5	9.6	89.4	30.3	90.5

Supplementary Table 2. Quinoa QQ74 Hi-C assembly statistics.

	PB	PB+BN	PB+Chi
Number of scaffolded contigs (% of total)	1,434 (31.04)	986 (22.71)	223 (7.09)
Length of scaffolded contigs, Gb (% of total)	1.202 (90.73%)	1.241 (89.58)	1.201 (90.53)
Number of scaffolded contigs with high-differential log-likelihood (% of total)	1240 (26.84)	919 (21.17)	214 (6.80)
Length of scaffolded contigs with high-differential log-likelihood, Gb (% of total)	1.180 (89.04)	1.221 (88.16)	1.197 (90.25)

Supplementary Table 3. Quantification of the number of collinear genes within and between different assemblies.

	Self comparisons				*B. vulgaris* comparisons			
	V1	PB	PB+BN	PB+Chi	V1	PB	PB+BN	PB+Chi
Analyzed genes	42,240	43,400	42,902	43,652	69,654	70,814	70,316	71,066
Collinear genes	25,845	27,845	27,379	28,227	29,341	30,234	30,130	30,492
Collinear genes (%)	61.19	64.16	63.82	64.66	42.12	42.69	42.85	42.91

Supplementary Table 4. Evaluation of V1, QQ74-V2, and C. suecicum V2 annotation completeness with BUSCO.

	V1	QQ74_V2	*C. suecicum* V2
Complete BUSCOs (%)	1,312 (95.4)	1,326 (96.4)	1,269 (92.3)
Complete and single-copy BUSCOs (%)	359 (26.1)	350 (25.5)	1,251 (91.0)
Complete and duplicated BUSCOs (%)	953 (69.3)	976 (71.0)	18 (1.3)
Fragmented BUSCOs (%)	35 (2.5)	31 (2.3)	59 (4.3)
Missing BUSCOs (%)	28 (2.0)	18 (1.3)	47 (3.4)
Total BUSCO groups searched	1,375	1,375	1,375

Supplementary Table 5. Comparison of PB+BN+Chi and QQ74_V2 gene models with GffCompare utility.

		No. of genes involved	
	Matches between PB+BN+Chi and QQ74-V2	V1	QQ74-V2
Total (% of total genes on QQ74-V2 pseudomolecules)	42,793	38,464 (88.1)	42,688 (80.8)
Intron chain compatible (%)	16,224 (37.9)	16,166 (42.0)	16,181 (37.9)
Complete, exact match of intron chain	10,151	10,145	10,145
Retained intron(s), all introns matched or retained	2,674	2,665	2,665
Containement of reference	2,525	2,499	2,500
Containement in reference	874	857	871
At least one junction match (%)	21,339 (49.9)	20,030 (52.1)	21,327 (50.0)
Multi-exon with at least one junction match	18,845	17,562	18,845
Retained intron(s), not all introns matched/covered	2,494	2,468	2,482
Other (%)	5,230 (12.2)	5,004 (13.0)	5,204 (12.2)

Supplementary Methods

The following scripts and commands provide additional details to supplement the information provided in the Methods section.

Gap filling with PBJelly

```
#!/bin/bash

#SBATCH --time=7-00:00:00   # walltime
#SBATCH --ntasks=32   # number of processor cores (i.e. tasks)
#SBATCH --nodes=1   # number of nodes
#SBATCH --mem-per-cpu=16384M   # memory per CPU core

module purge
module load pbsuite/15.8.24

Jelly.py setup Protocol.xml \
&& Jelly.py mapping Protocol.xml \
&& Jelly.py support Protocol.xml -x "--capturedOnly --spanOnly" \
&& Jelly.py extraction Protocol.xml \
&& Jelly.py assembly Protocol.xml -x "--nproc=24" \
&& Jelly.py output Protocol.xml -x "-m 2"

#you need to have a jellyProtocol.xml in the folder for this script to run correctly.  Here is an example of a Protocol.xml:
<jellyProtocol>
   <reference>lambda.fasta</reference>
   <outputDir>output_test</outputDir>
   <blasr>-minMatch 8 -minPctIdentity 70 -bestn 1 -nCandidates 20 -maxScore -500 -nproc 16 -noSplitSubreads</blasr>
   <input baseDir="/pbjelly/reads/">
      <job>filtered_subreads.fastq</job>
   </input>
</jellyProtocol>
```

Polishing with Pilon

```
#!/bin/bash

#SBATCH --time=72:00:00   # walltime
#SBATCH --ntasks=1   # number of processor cores (i.e. tasks)
#SBATCH --nodes=1   # number of nodes
#SBATCH --mem-per-cpu=512G   # memory per CPU core
#SBATCH -J "pilon"   # job name

reference_genome=asm.fasta
sorted_bam1=1.bam.sorted
sorted_bam2=2.bam.sorted #remove this line here and in the command below if you only have one bam.sorted file (you can also add additional bam.sorted files in a similar way)
output_dir=output
output_prefix=quinoa_pb_chicago-2-final_PBJELLY_pilon

module purge
module load conda/pilon_1.22

pilon -Xmx512G --genome ${reference_genome} --bam ${sorted_bam1} --bam ${sorted_bam2} --outdir ${output_dir} --output ${output_prefix} --changes --fix bases --diploid --threads 1
```

Gene and repeat annotation

1. RepeatModuler

```
#!/bin/bash

#SBATCH --time=168:00:00   # walltime
#SBATCH --ntasks=36   # number of processor cores (i.e. tasks)
#SBATCH --nodes=1   # number of nodes
#SBATCH --mem-per-cpu=16G   # memory per CPU core
#SBATCH -J "RepeatModeler"   # job name

module purge
module load repeatmodeler/1.0.11

name=asm
fasta=asm.fasta

BuildDatabase -name ${name} -engine ncbi ${fasta} && RepeatModeler -engine ncbi -pa 36 -database ${name}
```

2. RepeatMasker

```
#!/bin/bash

#SBATCH --time=72:00:00   # walltime
#SBATCH --ntasks=128   # number of processor cores (i.e. tasks)
#SBATCH --nodes=1   # number of nodes
#SBATCH --mem-per-cpu=4G   # memory per CPU core
#SBATCH -J "RepeatMasker"   # job name

module purge
module load RepeatMasker-4.1.2/4.1.2
module load python/3.8

lib=consensi.fa.classified #comes from RepeatModuler
fasta=asm.fasta
output=./repeatmasker_consensi_classified_align
#buildSummary.pl is in the util directory of RepeatMasker and may require a full path.

RepeatMasker -pa 128 -lib ${lib} -no_is -a -gff -dir ${output} ${fasta} && buildSummary.pl ${output}/${fasta}.out >
${output}/${fasta}.detailed.tbl
```

3. Gene annotation with MAKER

```
#!/bin/bash

#SBATCH --time=168:00:00   # walltime
#SBATCH --ntasks=8   # number of processor cores (i.e. tasks)
#SBATCH --nodes=1   # number of nodes
#SBATCH --mem-per-cpu=8G   # memory per CPU core

module purge
module load gcc/8 mpich/3.3 perl/5.28 bioperl/1.7 blast/2.9 snap-hmm/2019 repeatmasker/4.1 exonerate/2.2 maker/3.01-beta
module load evm_rapsearch_2

data_dir=./DATA #this is the directory houses the files needed for your annotation (e.g., transcriptome files, protein files, consensi
(repeatmasking) file, etc.
transcriptome=flnc_polished.hq.fasta # primary transcriptome
uniprot_sprot=uniprot_sprot.fasta # the latest uniprot_sprot.fasta
consensi=consensi.fa.classified # Output from RepeatModelers for species specific repeats
te_proteins=te_proteins.fa # provided by MAKER - probably not focused on plant repeat, but I usually include it regardless.
```

busco_augustus_directory=BUSCO_c_quinoa_asm.fasta-embryophyta_odb10-arabidopsis-long # this is the AUGUSTUS species specific model produced by BUSCO
alt1_EST=Bvulgaris_548_EL10_1.0.cds.fa
alt1_protein=Bvulgaris_548_EL10_1.0.protein.fa
snap=./snap/HMM/A.thaliana.hmm

maker -fix_nucleotide -c 8 ${scripts_dir}/${name}/maker_opts.ctl ${scripts_dir}/${name}/maker_bopts.ctl ${scripts_dir}/${name}/maker_exe.ctl

---

Maker requires three control files, which should be placed in the run directory:

1) maker_exe.ctl

```
#-----Location of Executables Used by MAKER/EVALUATOR
makeblastdb=/ MAKER_2.31.10/bin/makeblastdb #location of NCBI+ makeblastdb executable
blastn=/MAKER_2.31.10/bin/blastn #location of NCBI+ blastn executable
blastx=/MAKER_2.31.10/bin/blastx #location of NCBI+ blastx executable
tblastx=/MAKER_2.31.10/bin/tblastx #location of NCBI+ tblastx executable
formatdb= #location of NCBI formatdb executable
blastall= #location of NCBI blastall executable
xdformat= #location of WUBLAST xdformat executable
blasta= #location of WUBLAST blasta executable
exonerate=/apps/exonerate/2.2.0/gcc-8.3.0/bin/exonerate #location of exonerate executable
prerapsearch=/evm_rapsearch_2/bin #location of prerapsearch executable
rapsearch= /evm_rapsearch_2/bin #location of rapsearch executable
RepeatMasker=/repeatmasker/4.1.0/perl-5.28.0/RepeatMasker #location of RepeatMasker executable

#-----Ab-initio Gene Prediction Algorithms
snap=/snap-hmm/2019-06-03/gcc-9.2.0/bin/snap #location of snap executable
gmhmme3= #location of eukaryotic genemark executable
gmhmmp= #location of prokaryotic genemark executable
augustus= /MAKER_2.31.10/bin/augustus #location of augustus executable
fgenesh= #location of fgenesh executable
evm= /evm_rapsearch_2/bin/evidence_modeler.pl #location of EvidenceModeler executable
tRNAscan-SE=/MAKER_2.31.10/bin/tRNAscan-SE #location of trnascan executable
snoscan=/MAKER_2.31.10/bin/snoscan #location of snoscan executable


#-----Other Algorithms
probuild= #location of probuild executable (required for genemark)
```

2) maker_bopts.ctl

```
#-----BLAST and Exonerate Statistics Thresholds
blast_type=ncbi+ #set to 'ncbi+', 'ncbi' or 'wublast'
use_rapsearch=0 #use rapsearch instead of blastx, 1 = yes, 0 = no

pcov_blastn=0.8 #Blastn Percent Coverage Threhold EST-Genome Alignments
pid_blastn=0.85 #Blastn Percent Identity Threshold EST-Genome Aligments
eval_blastn=1e-10 #Blastn eval cutoff
bit_blastn=40 #Blastn bit cutoff
depth_blastn=0 #Blastn depth cutoff (0 to disable cutoff)

pcov_blastx=0.5 #Blastx Percent Coverage Threhold Protein-Genome Alignments
pid_blastx=0.4 #Blastx Percent Identity Threshold Protein-Genome Aligments
eval_blastx=1e-06 #Blastx eval cutoff
bit_blastx=30 #Blastx bit cutoff
depth_blastx=0 #Blastx depth cutoff (0 to disable cutoff)

pcov_tblastx=0.8 #tBlastx Percent Coverage Threhold alt-EST-Genome Alignments
pid_tblastx=0.85 #tBlastx Percent Identity Threshold alt-EST-Genome Aligments
eval_tblastx=1e-10 #tBlastx eval cutoff
bit_tblastx=40 #tBlastx bit cutoff
```

depth_tblastx=0 #tBlastx depth cutoff (0 to disable cutoff)

pcov_rm_blastx=0.5 #Blastx Percent Coverage Threhold For Transposable Element Masking
pid_rm_blastx=0.4 #Blastx Percent Identity Threshold For Transposbale Element Masking
eval_rm_blastx=1e-06 #Blastx eval cutoff for transposable element masking
bit_rm_blastx=30 #Blastx bit cutoff for transposable element masking

ep_score_limit=20 #Exonerate protein percent of maximal score threshold
en_score_limit=20 #Exonerate nucleotide percent of maximal score threshold

3) maker_opts.ctl

#-----Genome (these are always required)
genome=asm.fasta #genome sequence (fasta file or fasta embeded in GFF3 file)
organism_type=eukaryotic #eukaryotic or prokaryotic. Default is eukaryotic

#-----Re-annotation Using MAKER Derived GFF3
maker_gff= #MAKER derived GFF3 file
est_pass=0 #use ESTs in maker_gff: 1 = yes, 0 = no
altest_pass=0 #use alternate organism ESTs in maker_gff: 1 = yes, 0 = no
protein_pass=0 #use protein alignments in maker_gff: 1 = yes, 0 = no
rm_pass=0 #use repeats in maker_gff: 1 = yes, 0 = no
model_pass=0 #use gene models in maker_gff: 1 = yes, 0 = no
pred_pass=0 #use ab-initio predictions in maker_gff: 1 = yes, 0 = no
other_pass=0 #passthrough anyything else in maker_gff: 1 = yes, 0 = no

#-----EST Evidence (for best results provide a file for at least one)
est=$transcriptome} #set of ESTs or assembled mRNA-seq in fasta format
altest=${alt1_EST} #EST/cDNA sequence file in fasta format from an alternate organism
est_gff=
altest_gff= #aligned ESTs from a closly relate species in GFF3 format

#-----Protein Homology Evidence (for best results provide a file for at least one)
protein=${alt1_protein},${uniprot_sprot} #protein sequence file in fasta format (i.e. from mutiple oransisms)
protein_gff= #aligned protein homology evidence from an external GFF3 file

#-----Repeat Masking (leave values blank to skip repeat masking)
model_org= #select a model organism for RepBase masking in RepeatMasker
rmlib=${consensi} #provide an organism specific repeat library in fasta format for RepeatMasker
repeat_protein=${te_proteins} #provide a fasta file of transposable element proteins for RepeatRunner
rm_gff= #pre-identified repeat elements from an external GFF3 file
prok_rm=0 #forces MAKER to repeatmask prokaryotes (no reason to change this), 1 = yes, 0 = no
softmask=1 #use soft-masking rather than hard-masking in BLAST (i.e. seg and dust filtering)

#-----Gene Prediction
snaphmm=${snaphmm} #SNAP HMM file
gmhmm= #GeneMark HMM file
augustus_species=${busco_augustus_directory} #Augustus gene prediction species model
fgenesh_par_file= #FGENESH parameter file
pred_gff= #ab-initio predictions from an external GFF3 file
model_gff= #annotated gene models from an external GFF3 file (annotation pass-through)
run_evm=1 #run EvidenceModeler, 1 = yes, 0 = no
est2genome=1 #infer gene predictions directly from ESTs, 1 = yes, 0 = no
protein2genome=1 #infer predictions from protein homology, 1 = yes, 0 = no
trna=1 #find tRNAs with tRNAscan, 1 = yes, 0 = no
snoscan_rrna= #rRNA file to have Snoscan find snoRNAs
snoscan_meth= #-O-methylation site fileto have Snoscan find snoRNAs
unmask=0 #also run ab-initio prediction programs on unmasked sequence, 1 = yes, 0 = no
allow_overlap= #allowed gene overlap fraction (value from 0 to 1, blank for default)

#-----Other Annotation Feature Types (features MAKER doesn't recognize)

```
other_gff= #extra features to pass-through to final MAKER generated GFF3 file

#-----External Application Behavior Options
alt_peptide=C #amino acid used to replace non-standard amino acids in BLAST databases
cpus=8 #max number of cpus to use in BLAST and RepeatMasker (not for MPI, leave 1 when using MPI)

#-----MAKER Behavior Options
max_dna_len=100000 #length for dividing up contigs into chunks (increases/decreases memory usage)
min_contig=500 #skip genome contigs below this length (under 10kb are often useless)

pred_flank=200 #flank for extending evidence clusters sent to gene predictors
pred_stats=0 #report AED and QI statistics for all predictions as well as models
AED_threshold=1 #Maximum Annotation Edit Distance allowed (bound by 0 and 1)
min_protein=0 #require at least this many amino acids in predicted proteins
alt_splice=0 #Take extra steps to try and find alternative splicing, 1 = yes, 0 = no
always_complete=0 #extra steps to force start and stop codons, 1 = yes, 0 = no
map_forward=0 #map names and attributes forward from old GFF3 genes, 1 = yes, 0 = no
keep_preds=0 #Concordance threshold to add unsupported gene prediction (bound by 0 and 1)

split_hit=10000 #length for the splitting of hits (expected max intron size for evidence alignments)
single_exon=1 #consider single exon EST evidence when generating annotations, 1 = yes, 0 = no
single_length=250 #min length required for single exon ESTs if 'single_exon is enabled'
correct_est_fusion=0 #limits use of ESTs in annotation to avoid fusion genes

tries=3 #number of times to try a contig if there is a failure for some reason
clean_try=1 #remove all data from previous run before retrying, 1 = yes, 0 = no
clean_up=0 #removes theVoid directory with individual analysis files, 1 = yes, 0 = no
TMP= #specify a directory other than the system default temporary directory for temporary files
```

Assigning functionality to the predicted gene models using homology to uniprot-sprot reference protein database

1. Blast predicted proteins against uniprot-sprot database

```
#!/bin/bash

#SBATCH --time=12:00:00   # walltime
#SBATCH --ntasks=28   # number of processor cores (i.e. tasks)
#SBATCH --nodes=1   # number of nodes
#SBATCH --mem-per-cpu=20G   # memory per CPU core

module purge
module load gcc/8 mpich/3.3 perl/5.28 bioperl/1.7 blast/2.9 snap-hmm/2019 repeatmasker/4.1 exonerate/2.2 maker/3.01-beta

#you need to make a blastdb for the uniprot_sprot.fasta.  GET the latest version from:
ftp://ftp.uniprot.org/pub/databases/uniprot/current_release/knowledgebase/complete/uniprot_sprot.fasta.gz

data_dir=/maker/DATA

#makeblastdb -in ${data_dir}/uniprot_sprot.fasta -input_type fasta -dbtype prot ##if you need to make a blastdb
blastp -db uniprot_sprot.fasta -query maker.proteins.fasta -out maker2uni.blasp -evalue 0.000001 -outfmt 6 -num_alignments 1 -seg yes -soft_masking true -lcase_masking -max_hsps 1 -num_threads $SLURM_NPROCS
```

2. Use maker script to add functionality to description of gene models in gff as well as proteins.fasta and transcripts.fasta files

```
#!/bin/bash

#SBATCH --time=12:00:00   # walltime
```

```
#SBATCH --ntasks=2   # number of processor cores (i.e. tasks)
#SBATCH --nodes=1   # number of nodes
#SBATCH --mem-per-cpu=4G   # memory per CPU core

data_dir=/maker/DATA/uniprot

module purge
module load gcc/8 mpich/3.3 perl/5.28 bioperl/1.7 blast/2.9 snap-hmm/2019 repeatmasker/4.1 exonerate/2.2 maker/3.01-beta

maker_functional_gff ${data_dir}/uniprot_sprot.fasta uniprot_sprot_maker2uni.blasp Final_allscaffolds.gff > Final.maker.functional.gff &&

maker_functional_fasta ${data_dir}/uniprot_sprot.fasta uniprot_sprot_maker2uni.blasp maker.proteins.fasta >
Final.maker.proteins_functional_blast.fasta &&

maker_functional_fasta ${data_dir}/uniprot_sprot.fasta uniprot_sprot_maker2uni.blasp maker.transcripts.fasta >
Final.maker.transcripts_functional_blast.fasta
```

Assessing assembly completeness with BUSCO

```
#!/bin/bash

#SBATCH --time=72:00:00   # walltime
#SBATCH --ntasks=36   # number of processor cores (i.e. tasks)
#SBATCH --nodes=1   # number of nodes
#SBATCH --mem-per-cpu=15G   # memory per CPU core
#SBATCH -J "busco5.0"   # job name

module purge
module load busco_5.0

assembly=assembly.fa
output_dir= /busco5.0_outputs
lineage=embryophyta_odb10 #or use any odb10 database of interest – download from https://busco.ezlab.org
augustus_species=Arabidopsis
download_path=/busco5.0_downloads

busco -i ${assembly} -l ${lineage} -o ${assembly}-${lineage}-${augustus_species}-long -m genome -c $SLURM_NPROCS --out_path ${output_dir}
--download_path ${download_path} --augustus --augustus_species ${augustus_species} --long -f --offline
```

Assessing assembly completeness with LAI score analysis

```
##Using LTR_retriever (v2.8.7)
time gt -j 64 ltrharvest -index QQ74_V2.fasta -minlenltr 100 -maxlenltr 7000 -mintsd 4 -maxtsd 6 -motif TGCA -motifmis 1 -similar 85 -vic 10 -seed
20 -seqids yes >QQ74_V2.harvest.scn
time ~/softw/LTR_FINDER_parallel/LTR_FINDER_parallel -seq QQ74_V2.fasta -threads 64 -harvest_out -size 1000000

cat QQ74_V2.fasta.finder.combine.scn QuinoaV2.harvest.scn >QQ74_V2.rawLTR.scn
time LTR_retriever -genome QQ74_V2.fasta -inharvest QQ74_V2.rawLTR.scn -threads 64
#results in 1st line of QQ74_V2.fasta.out.LAI
# The same command lines were applied to QQ74 V1 and C. suecicum V2 assemblies.
```

Assessing assembly completeness with K-mer analysis

```
for i in $(ls *.gz); do out=${i/.fastq.gz/}; time meryl k=19 count output $out.mery $i; done
meryl union-sum output quinoa.meryl *.mery
merqury.sh ./illumina/quinoa.meryl ../LAI/V1/CQ41.fasta ../LAI/V2/Csuecicum_V2.fasta ../LAI/V2/QuinoaV2.fasta Quinoa_merqury
#results of completeness were collected from "Quinoa_merqury.completeness.stats" and QV scores from "Quinoa_merqury.qv"
```

Assessing assembly completeness with Illumina read mapping

###Using bowtie2 (v2.5.1) and samtools (v1.7)
###Mapping C. quinoa QQ74 illumina reads (SRR3740394) against QQ74_V1 (here CQ41.fasta) and QQ74_V2 (here quinoa_pb_chicago-2-final_PBJELLY_pilon_pseudo_RENAMED.fasta) genome assemblies.

```
bowtie2-build CQ41.fasta CQ41
bowtie2 -x CQ41 -1 SRR3740394_1.fastq.gz -2 SRR3740394_2.fastq.gz --sensitive -p 20 >illumina-vs-CQ41_sensitive.bam

bowtie2-build quinoa_pb_chicago-2-final_PBJELLY_pilon_pseudo_RENAMED.fasta quinoa_pb_chicago-2-final_PBJELLY_pilon_pseudo_RENAMED
bowtie2 -x quinoa_pb_chicago-2-final_PBJELLY_pilon_pseudo_RENAMED -1 SRR3740394_1.fastq.gz -2 SRR3740394_2.fastq.gz --sensitive -p 20 >illumina-vs-QQ74_V2_sensitive.bam
```

###Mapping C. suecicum illumina reads (SRR4425238) against C. suecicum V2 (here Csuecicum_v2_pseudoonly.fasta) genome assembly.

```
bowtie2-build /data/ManuscriptV2/mappingCsuecicum/Csuecicum_v2_pseudoonly.fasta Csuecicum_v2_pseudoonly
```

Assessing gene annotation quality and completeness

##BUSCO analysis on peptides
BUSCO v4.0.4
lineage: embryophyta_odb10 (Creation date: 2017-12-01, number of species: 60, number of BUSCOs: 1375)
BUSCO was run in mode: protein
E-value cutoff was set to 10e-5.

##AGAT stats on annotations

Another GFF Analysis Toolkit (AGAT) - Version: v0.5.1
  https://github.com/NBISweden/AGAT
  National Bioinformatics Infrastructure Sweden (NBIS) - www.nbis.se

```
agat_sp_statistics.pl --gff file.gff
```

Visualizing genome features in Circos plots

##Make 1-Mb windows maps

##Create a file genome.length with chr_start_stop information for each genome:

```
cat Cq_chr.length
Cq1A	1	57138015
Cq1B	1	71683396
…
```

##Make 1Mb genome windows file

```
bedtools makewindows –g Cq_chr.length -w 1000000 > Cq_1M.wind.sorted
```

--> Do the same for each *C. quinoa*, *C. pallidicaule*, and *C. suecicum* genomes.

##REPEAT density for circos
using bedtools v2.29.1

```
cat Cquinoa_REPETfull_pseudo_RENAMED_matchonly.gff3 | cut -f 1,4,5 >REPETfull.pos
bedtools sort -i REPETfull.pos >REPETfull.pos.sorted
bedtools merge -i REPETfull.pos.sorted >REPETfull.pos.sorted.merged
awk '{print $1"\t"$2"\t"$3"\t"$3-$2}' REPETfull.pos.sorted.merged >Repeat.sized
awk '{print $1"\t"$2"\t"$3"\tmap-"NR"\t"$3-$2}' Repeat.sized >REPETfull.bedmap
bedtools map -delim '\t' -o sum -a Cq_1M.wind.sorted -b REPETfull.bedmap | sed -re 's/.000000$//g' >Cq_1M.repeats.density
```

--> Do the same for each *C. quinoa*, *C. pallidicaule*, and *C. suecicum* genomes.

```
##GENE density for circos
using bedtools v2.29.1
cat Cquinoa_pseudo_RENAMED_matchonly.gff3 | cut -f 1,4,5 >genes.pos
bedtools sort -i genes.pos >genes.pos.sorted
awk '{print $1"\t"$2"\t"$3"\tmap-"NR"\t"$3-$2}' genes.pos.sorted > genes.bedmap
bedtools map -delim '\t' -o sum -a Cq_1M.wind.sorted -b genes.bedmap | sed -re 's/.000000$//g' >Cq_1M.genes.density
```

--> Do the same for each *C. quinoa*, *C. pallidicaule*, and *C. suecicum* genomes.

```
##Make collinearity file

!/bin/bash
makeblastdb -in Bv_Cp_Cq_Cs.pep -dbtype prot -parse_seqids
blastp -task blastp -query Bv_Cp_Cq_Cs.pep -db Bv_Cp_Cq_Cs.pep -out blast_Bv_Cp_Cq_Cs_pep-itself -num_threads 40 -outfmt 6 -num_descriptions 5 -num_alignments 5 -evalue 1e-10
--> this produces the blast_Bv_Cp_Cq_Cs_pep-itself.blast file
--> add blast_Bv_Cp_Cq_Cs_pep-itself.blast & Bv_Cp_Cq_Cs.gff files into Synteny folder
--> where Bv_Cp_Cq_Cs.gff has 4 columns: ‘SpeciesChromosome#_geneID_start_stop’
##using MCScanX
./MCScanX Synteny/ Bv_Cp_Cq_Cs –s 8
head Bv_Cp_Cq_Cs.collinearity
############### Parameters ###############
# MATCH_SCORE: 50
# MATCH_SIZE: 8
# GAP_PENALTY: -1
# OVERLAP_WINDOW: 5
# E_VALUE: 1e-05
# MAX GAPS: 25
############### Statistics ###############
# Number of collinear genes: 77376, Percentage: 68.60
# Number of all genes: 112799
##########################################
## Alignment 0: score=3374.0 e_value=1.5e-266 N=69 Bv1&Cp1 plus
  0-  0:	KMT20634	CP014306-RA	      0
  0-  1:	KMT20635	CP014307-RA	      0
  0-  2:	KMT20636	CP014309-RA	  3e-52
  0-  3:	KMT20637	CP014310-RA	      0
  0-  4:	KMT20638	CP014311-RA	      0
  0-  5:	KMT20639	CP014313-RA	  7e-34

cat Bv_Cp_Cq_Cs.collinearity | grep –v “^#” | cut –f 2 >list.1
cat Bv_Cp_Cq_Cs.collinearity | grep –v “^#” | cut –f 3 >list.2
for i in $(cat list.1); do grep –w “$i"  Bv_Cp_Cq_Cs.gff | cut –f 1,3,4 >list1.tab
for i in $(cat list.2); do grep –w “$i"  Bv_Cp_Cq_Cs.gff | cut –f 1,3,4 >list2.tab
paste list1.tab list2.tab > Cq_itself_collinearity_8genes_circos.csv
```

--> manually edit Cq_itself_collinearity_8genes_circos.csv to add a 7th column with color and thickness information for the circos links.

##Make the diploid reads mapping tracks

bowtie2-build quinoa_pb_chicago-2-final_PBJELLY_pilon_pseudo_RENAMED.fasta quinoa_pb_chicago-2-final_PBJELLY_pilon_pseudo_RENAMED

bowtie2 -x quinoa_pb_chicago-2-final_PBJELLY_pilon_pseudo_RENAMED -1 diploidreads_1.fastq.gz -2 diploidreads_2.fastq.gz --sensitive -p 20 >diploid-vs-QQ74_V2_sensitive.bam

##diploid reads are *C. pallidicaule* (SRR4425239) and *C. suecicum* (SRR4425238).

mosdepth -n –q 20 -t 40 -b Cq_1M.wind.sorted *diploid-vs-QQ74_V2_*Cq_1M.wind *diploid-vs-QQ74_V2_sensitive.bam*

gunzip *diploid-vs-QQ74_V2_*Cq_1M.wind.regions.bed.gz

Identifying discordant mapping reads at Cq3B inversion breakpoints

--> Nb. Of discordant reads at Cq3B:11,136,405
for i in $(ls *.bam); do echo "$i"; samtools view $i Cq3B:11136100-11137000 | awk '($9>=52223700 && $9<=52225600) {print}' | wc -l >$i.11136100-11137000.tab; done
for i in $(ls *11137000.tab); do echo "$i"; cat $i ;done >>Cq3B_11136100-11137000-discordant.txt
--> Nb. Of discordant reads at Cq3B:63,361,214
for i in $(ls *.bam); do echo "$i"; samtools view $i Cq3B:63360700-63361700 | cut -f 9 | sed 's/-//g' | awk '($1>=52223700 && 19<=52225600) {print}' | wc -l >$i.63360700-63361700.tab; done
for i in $(ls *63361700.tab); do echo "$i"; cat $i ;done >>Cq3B_63360700-63361700-discordant.txt

Assessing distribution of the Cq3B inversion within quinoa diversity panel

##Produce PHYLIP format file from SNPs
/data/software/vcf2phylip-1.5/vcf2phylip.py --input Cq_allpseudo_biallelic_minDP5_MaxMissing0.8_MAF0.01.Cq3Bsamples.variantsonly.vcf.recode.vcf

###Build the tree
iqtree -s Cq_allpseudo_biallelic_minDP5_MaxMissing0.8_MAF0.01.Cq3Bsamples.variantsonly.min4.recode.min4.phy -m GTR+F+R8 -B 1000 -T 60

###Visuallize the tree
https://itol.embl.de/
