# Supplementary material for: A chromosome-scale assembly of the quinoa genome provides insights into the structure and dynamics of its subgenomes
Source: Commun Biol. 2023 Dec 13;6:1263. doi: 10.1038/s42003-023-05613-4 (PMC10719370; doi:10.1038/s42003-023-05613-4)
Supplement: Supplementary file 4 — Description of Additional Supplementary Files [file 42003_2023_5613_MOESM4_ESM.pdf]

Description of Additional Supplementary Files

File name: Supplementary Data 1
Description: Correspondence table of gene IDs from the quinoa V1 and QQ74-V2 annotations.

File name: Supplementary Data 2
Description: Repetitive elements annotation statistics

File name: Supplementary Data 3
Description: Representation of complete LTR element families between C. quinoa subgenomes

File name: Supplementary Data 4
Description: Repeat content and dynamics between genomes

File name: Supplementary Data 5
Description: Scoring the Cq3B pericentromeric inversion in 184 quinoa accessions

File name: Supplementary Data 6
Description: Syntenic relationships between quinoa subgenomes

File name: Supplementary Data 7
Description: Chromosomal rearrangements within and between C. quinoa subgenomes

File name: Supplementary Data 8
Description: Patterns of diploid C. pallidicaule and C. suecicum read mapping against quinoa subgenomes
